# Red Yeast Rice Preparations Reduce Mortality, Major Cardiovascular Adverse Events, and Risk Factors for Metabolic Syndrome: A Systematic Review and Meta−analysis

**DOI:** 10.3389/fphar.2022.744928

**Published:** 2022-02-21

**Authors:** Rong Yuan, Yahui Yuan, Lidan Wang, Qiqi Xin, Ya Wang, Weili Shi, Yu Miao, Sean Xiao Leng, Keji Chen, Weihong Cong, and BPNMI Consortium

**Affiliations:** ^1^ Laboratory of Cardiovascular Diseases, Xiyuan Hospital, China Academy of Chinese Medical Sciences, Beijing, China; ^2^ National Clinical Research Center for Chinese Medicine Cardiology, Xiyuan Hospital, China Academy of Chinese Medical Sciences, Beijing, China; ^3^ Division of Geriatric Medicine and Gerontology, Department of Medicine, Johns Hopkins University School of Medicine, Baltimore, MD, United States

**Keywords:** red yeast rice, metabolic syndrome, clinical endpoints, risk factors, dyslipidaemia, hypertension, diabetes

## Abstract

**Background:** Metabolic syndrome (MetS) is characterized by the cooccurrence of obesity, insulin resistance, dyslipidaemia, and hypertension. Red yeast rice (RYR) preparations might be beneficial for the prevention and treatment of MetS.

**Objective:** To implement a systematic review and meta−analysis to determine whether RYR preparations improve clinical endpoints and reduce risk factors for MetS.

**Methods:** The PubMed, Cochrane Library, EMBASE, Scopus, China National Knowledge Infrastructure, Chinese VIP Information, and WanFang databases were searched for randomized controlled trials (published up to September 2020), and a meta−analysis was performed using fixed− or random−effects models. The primary outcome measures were mortality and major adverse cardiovascular events (MACEs), and the secondary outcome measures were biochemical parameters of blood glucose, blood lipids, and blood pressure. The registration number is CRD42020209186.

**Results:** A total of 921 articles were identified, of which 30 articles were included in this article. RYR preparations group demonstrated significant improvements in MetS compared with control group. RYR preparations reduced the mortality and MACEs (RR = 0.62, 95% CI [0.49, 0.78]; RR = 0.54, 95% CI [0.43, 0.66]). In terms of blood glucose metabolism, fasting plasma glucose (FPG) (MD = −0.46 mmol/L, 95% CI [−0.71, −0.22]), haemoglobin A1c (HbA1c) (MD = −0.49, 95% CI [−0.71, −0.26]) and the homeostasis model assessment of insulin resistance (HOMA−IR) (MD = −0.93, 95% CI [−1.64, −0.21]) were decreased. Regarding the lipid metabolism, total cholesterol (TC) (MD = −0.74 mmol/L, 95% CI [−1.02, −0.46]), triglycerides (TG) (MD = −0.45 mmol/L, 95% CI [−0.70, −0.21]), and low−density lipoprotein cholesterol (LDL) (MD = −0.42 mmol/L, 95% CI [−0.78, −0.06]) were decreased, while high−density lipoprotein cholesterol (HDL) (MD = 0.14 mmol/L, 95% CI [0.09, 0.20]) was increased. Regarding blood pressure, the mean arterial pressure (MAP) (MD = −3.79 mmHg, 95% CI [−5.01, −2.57]) was decreased. In addition, RYR preparations did not increase the incidence of adverse reactions (RR = 1.00, 95% CI [0.69, 1.43]).

**Conclusion:** RYR preparations reduce mortality, MACEs, and multiple risk factors for MetS without compromising safety, which supports its application for the prevention and treatment of MetS. However, additional high−quality studies are needed to provide more evidence for the effect of RYR on MetS due to the heterogeneity in this study.

**Systematic Review Registration**: www.crd.york.ac.uk/PROSPERO, identifier CRD42020209186

## Introduction

Metabolic syndrome (MetS) comprises atherogenic dyslipidaemia, hypertension, obesity, and insulin resistance (Hu et al., 2020), and is associated with an increased risk of type 2 diabetes mellitus, nonalcoholic fatty liver disease, myocardial infarction, and stroke (Prasun, 2020). MetS has become increasingly prevalent with the improvement of people’s lives, and it is associated with an increased risk for cardiovascular disease (CVD) and all−cause mortality (Hirode and Wong, 2020). At present, MetS is an urgent global health problem that is unresolved, and there is no single drug that simultaneously treats the multiple diseases or regulates complex underlying mechanisms of MetS yet (Hu et al., 2020). Therefore, it is important to identify therapies that impede the development of metabolic diseases from a preventive perspective.

Red yeast rice (RYR), also known as Hong Qu, Hon−Chi, Anka and red Koji, is a functional food containing monacolin K (lovastatin), monacolin KA, citrinin (ppb), and so on (Halber et al., 2010), and active ingredient monacolin K is the first statin drug (lovastatin) to be approved for treatment of high cholesterol levels (Hunter and Hegele, 2017). RYR is made by fermenting steamed rice with an edible fungus, *Monascus purpureus* Went. RYR products included RYR powders, dietary supplements, and Chinese proprietary medicines (Xuezhikang and Zhibituo), and ingredients were controllable (Klingelhöfer, and Morlock, 2019). Substantial evidence suggests a strong biochemical effect of RYR on plasma lipid levels and it is frequently consumed to lower low−density lipoprotein (LDL) as an alternative to statins (Loubser et al., 2019).

Several meta−analyses have shown that RYR is an effective and relatively safe therapy for dyslipidaemia (Xiong et al., 2017; Sungthong et al., 2020). Increasing evidence also suggests that RYR has antidyslipidaemia, antidiabetic, antiatherosclerotic, antiobesity, and antihypertensive activities (Zhu et al., 2019), leading to a growing interest in the hypothesis that RYR has beneficial health effects on MetS and its adverse clinical outcomes. Thus, we aimed to evaluate the efficacy and safety of RYR preparations in MetS through a systematic review and meta−analysis of randomized controlled trials (RCTs).

## Methods

This study was conducted and reported based on the guidelines of PRISMA and was registered in PROSPERO (the registration number is CRD42020209186).

### Search Strategies

We identified articles by searching the following electronic databases from database inception until September 2020: PubMed, Cochrane Library, EMBASE, Scopus, China National Knowledge Infrastructure, Chinese VIP Information, and WanFang. The search strategy included population and intervention keywords (MetS and RYR preparations) as well as synonyms of these terms (Supplementary Material S1). We also manually searched the reference lists of the included articles. Authors of relevant studies were contacted to obtain additional and missing data. Two reviewers (RY, YY) independently identified relevant studies, and any disagreement was resolved through discussion or consultation with a third reviewer.

### Selection of Studies

The inclusion criteria were as follows: a) Populations: participants meeting at least one of the international or domestic diagnostic criteria for MetS with no restrictions of age, sex, race, course; b) Interventions: patients in the treatment group were given RYR preparations alone or combined with conventional treatment (hypoglycemic drugs, lipid−lowering drugs, and antihypertensive drugs alone or the combination of these drugs), defining RYR preparations as involving Xuezhikang interventions or other relevant preparations; c) Comparators: patients in the control group were given conventional treatment or placebo; d) Outcomes: reported data for at least one of the interest outcomes: the primary outcomes of interest were mortality and MACEs, the secondary outcomes of interest included MetS risk factors, such as blood sugar parameters [fasting plasma glucose (FPG), haemoglobin A1c (HbA1c), homeostasis model assessment of insulin resistance (HOMA−IR), insulin sensitivity index (ISI)], blood lipid parameters [total cholesterol (TC), triglycerides (TG), LDL, high−density lipoprotein (HDL)], and blood pressure [mean arterial pressure (MAP), systolic blood pressure (SBP), and diastolic blood pressure (DBP)]. In addition, adverse reactions were also assessed; e) Study designs: RCTs.

The exclusion criteria were as follows: a) included patients with severe abnormal heart, liver and kidney function, severe infections, tumor or other serious primary disease; b) included patients with special populations such as lactating woman or pregnant women; c) patients in the treatment group were given nutraceuticals combination compound; d) no clinical data could be extracted; e) reviews, retrospective studies, observational studies, letters, case reports, and animal/cell experiments.

### Data Extraction

The Cochrane Data Collection form was applied for the data extraction. Two independent investigators (RY and YY) reviewed the studies and extracted the data. The following data were extracted for each study: participant characteristics, such as the number of participants in each group, age, sex, drug and control treatment, intervention duration, outcomes (major adverse cardiovascular events (MACEs), mortality, and the biochemical parameters of blood glucose, blood lipids, and blood pressure), and adverse events.

### Quality Assessment

Two reviewers (YR, YY) independently evaluated the quality of included studies according to the Physiotherapy Evidence Database (PEDro) scale (Moseley et al., 2020), which is exclusively applied to assess the risk of bias in RCTs. The PEDro criteria items include eligibility criteria, random allocation, concealed allocation, group similarity at baseline, participant/therapist/assessor blinding, dropout number, intention−to−treat analysis, between−group differences, and point estimate and variability. The PEDro scale awards 1 point per criterion (the first criterion is not scored): a score of less than 4 is considered “poor”, 4 to 5 is considered “fair”, 6 to 8 is considered “good” and 9 to 10 is considered ‘excellent’ (Cashin and Mcauley, 2020). In addition, we assessed the quality of included evidence using the Grading of Recommendations, Assessment, Development and Evaluations (GRADE) approach, which classifies the evidence as high, moderate, low, or very low.

### Data Analysis

RevMan 5.3.0 was utilized to analyse the results. Continuous variables were calculated by the mean difference (MD) and 95% confidence interval (CI). Dichotomous variables were expressed as relative risk (RR) with 95% CI. According to the Cochrane Handbook for Systematic Reviews of Interventions, once the multiple treatment groups were included from one study, the shared control group were split into two or more groups with smaller sample sizes, and two or more (reasonably independent) comparisons were included (Higgins et al., 2019). The heterogeneity of the results across the studies was evaluated using the *I* ^2^ statistic: *I* ^2^ < 50% were considered to have low heterogeneity and the results were estimated by a fixed−effects model, while *I* ^2^ ≥ 50% were considered to have moderate and high heterogeneity and the results were estimated by a random−effects model. In cases of heterogeneity, we investigated the potential causes by performing subgroup and meta−regression analyses. When there were high levels of heterogeneity, study characteristics and data−related factors (such as age, sex, intervention duration and intervention type) were explored to identify the cause of the heterogeneity. Stata 15.1 was utilized to draw funnel plots, and we conducted Egger’s test to detect publication bias when the number of studies for each outcome was above or equal to 10.

## Results

### Study Selection

**FIGURE 1:**
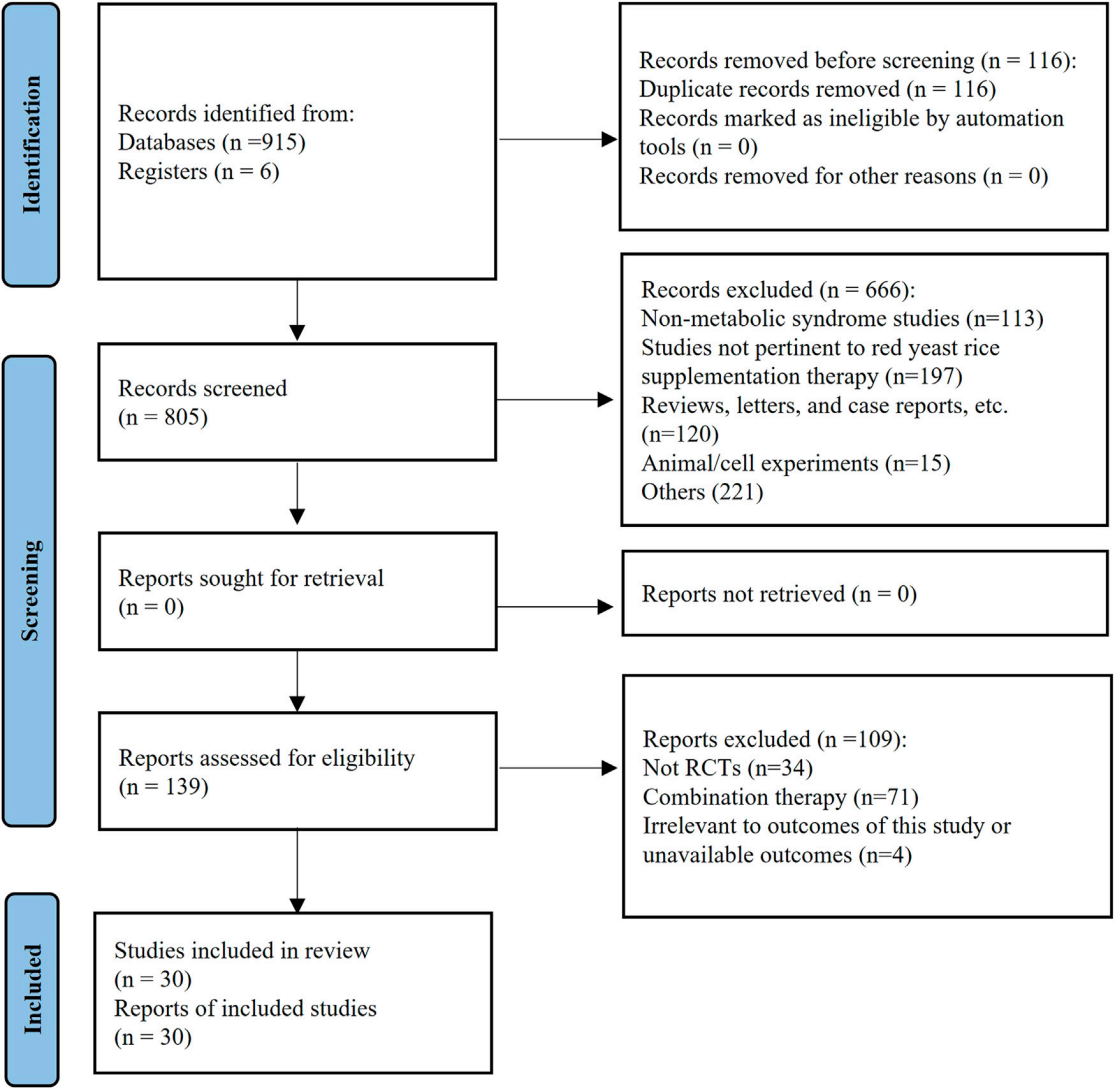
Overview of the systematic review process.

A total of 921 articles were searched, and 805 articles were screened by the title and abstract after the removal of duplicates. A total of 139 articles were identified for review based on the full text, and 30 articles were eligible to be included in the review (Figure 1). Data were only included once in meta−analysis when participant data reported in more than one publication.

### Study Characteristics

In this meta−analysis, Li et al., 2017 contained two treatment groups and presented the results separately for Xuezhikang or Xuezhikang + Daixiefang compared with Daixiefang (a traditional Chinese medicine prescription). Two articles (Li et al., 2010) (Li et al., 2009) reported data from one trial. The clinical studies were published in English and Chinese from 1997 to 2019, and all of the studies were conducted in China except for one from Italy. A total of 5440 patients were included, and the mean age of the participants ranged from 41 to 76. Interventions included Xuezhikang and lovastatin, and trial duration ranged from 1 month to 4.5 years. The detailed characteristics of the included trials are shown in Table 1. The detailed information of RYR preparations in the included trials is described in Supplementary Table S1. The PEDro score ranged from 4 (Shen, 2011; Zhang and Wu, 2014) to 9 (Li et al., 2009; Li et al., 2010; Li et al., 2017) (Supplementary Table S2).

**TABLE 1:** Study characteristics.

Study (country)	Population	PEDro score	Participants (int:cont)	Male: Female (int/cont)	Age range	Trial intervention	Control intervention	Period of treatment	Outcomes
Zhang, (2019)	Diabetes + hyperlipidaemia	6	67:67	33:34/36:31	int: 45.19 ± 3.84	Lovastatin + metformin	Metformin	8 weeks	TC, TG, FPG, HbA1c
					cont: 45.18 ± 1.91				
Xiao, (2019)	Hyperlipidaemia + prehypertension	6	42:42	23:19/25:17	int: 67.53 ± 5.76	Xuezhikang	Atorvastatin calcium	4 weeks	TC, TG, HDL−C, LDL−C SBP, DBP, MAP
					cont: 68.24 ± 6.13				
Li et al. (2017)	MetS	9	20:21	5:15/11:10	int: 68.25 ± 10.92/cont: 62.38 ± 9.90	Xuezhikang	Daixiefang^*^	12 weeks	TC, TG, HDL−C, LDL−C, FPG, HbA1c, HOMA−IR
			21:21	7:14/11:10	int: 66.10 ± 8.85	Xuezhikang + Daixiefang			
					cont: 62.38 ± 9.90				
Wang and Wang, (2016)	Hyperlipidaemia + prehypertension	6	62:62	48:14/46:16	int: 66.7 ± 7.9	Xuezhikang	Atorvastatin calcium	8 weeks	TC, TG, HDL−C, LDL−C, SBP, DBP, MAP
					cont: 67.6 ± 8.1				
Zhao, (2015)	Diabetes + dyslipidaemia	5	32:33	17:15/15:18	int: 59.6 ± 4.7	Xuezhikang	Placebo	8 weeks	TC, TG, HDL−C, LDL−C
					cont: 57.6 ± 5.3				
Ke, (2015)	Diabetes + dyslipidaemia	6	29:29	18:11/16:13	int: 62.3 ± 6.6	Xuezhikang	Simvastatin	12 weeks	TC, TG, HDL−C, LDL−C, FPG
					cont: 62.9 ± 7.1				
Zhang and Wu, (2014)	MetS	4	20:20	24:16	76 ± 10	Xuezhikang + routine treatment	Routine treatment	12 weeks	TG, HDL−C, SBP, DBP, FPG, HOMA−IR
Chen, (2013)	Diabetes + hyperlipidaemia	7	45:44	Not reported	45–70	Xuezhikang	Simvastatin	12 weeks	TC, TG, HDL−C, LDL−C
Wang and Liu, (2013)	Diabetes + dyslipidaemia	6	32:30	18:14/13:17	int: 59 ± 7.6	Xuezhikang	Simvastatin	8 weeks	TG
					cont: 61 ± 9.3				
Wu and Wei, (2011)	Hypertension + dyslipidaemia	5	75:75	48:27/49:26	int: 54.8 ± 10.2	Xuezhikang	Atorvastatin calcium	8 weeks	TC, TG, HDL−C, LDL−C
					cont: 55.26 ± 9.8				
Ma and Qian, (2011)	Diabetes + dyslipidaemia	6	37:37	22:15/24:13	int: 42–66	Xuezhikang	Simvastatin	6 weeks	TC, TG, HDL−C
					cont: 41–65				
Shen, (2011)	Diabetes + dyslipidaemia	4	40:25	35:30	45–65	Xuezhikang + routine treatment	Routine treatment	18 weeks	TG, HDL−C, LDL−C, MACEs
Li et al., 2010	Hypertension + dyslipidaemia	9	1363:1341	1093:270/1054:287	int: 59.4 ± 9.2	Xuezhikang	Placebo	4.5 years	TC, TG, HDL−C, LDL−C, SBP, DBP, mortality, MACEs
					cont: 59.2 ± 9.5				
Zhai and Li, (2010)	Diabetes + hyperlipidaemia	5	48:38	28:20/21:17	int: 50.6	Xuezhikang + routine treatment	Routine treatment	12 weeks	TG, TC
					cont: 51.8				
Li et al, (2009)	Hypertension + dyslipidaemia	9	772:758	1143/387	int: 66 ± 4	Xuezhikang	Placebo	4.5 years	SBP, DBP, TG, TC, LDL−C, HDL−C
					cont: 66 ± 4				
He and Zhan, (2009)	Diabetic nephropathy	6	72:72	56:16/58:14	int: 53.3 ± 9.4	Xuezhikang	Atorvastatin calcium	48 weeks	TC, TG
					cont: 51.3 ± 11.6				
Zhang, (2009)	Diabetes + dyslipidaemia	8	45:43	33:12/29:14	int: 61.2 ± 4.6	Xuezhikang + routine treatment	Routine treatment	16 weeks	TC, TG, HDL−C
					cont: 58.8 ± 6.2				
Jin et al. (2007)	Diabetic nephropathy + dyslipidaemia	6	30:30	21:9/19:11	int: 52.81 ± 9.39	Xuezhikang + routine treatment	Routine treatment	12 weeks	TC, TG, HDL−C, FPG
					cont: 54.96 ± 7.98				
Zhao et al. (2007)	Diabetes + dyslipidaemia	7	306:285	226:80/198:87	int: 60.5 ± 8.7	Xuezhikang	Placebo	4 years	TC, TG, HDL−C, LDL−C, mortality, MACEs
					cont: 61.6 ± 8.0				
Wang et al. (2005)	Diabetes + hyperlipidaemia	5	32:30	17:15/16:14	int: 54.2 ± 10.4	Xuezhikang	Pravastatin	8 weeks	TC, TG, HDL−C, LDL−C
					cont: 55.1 ± 9.5				
Zhu and Yao, (2005)	Diabetes + hyperlipidaemia	6	31:31	40:22	51–75	Xuezhikang	Routine treatment	4 weeks	TC, TG, FPG
Li et al. (2005)	Diabetes + hyperlipidaemia	6	24:24	Not reported	40–50	Xuezhikang + routine treatment	Routine treatment	8 weeks	TC, TG, HDL−C, LDL−C, FPG, HbA1c
Yao et al. (2004)	Diabetes + hyperlipidaemia	6	24:22	12:12/12:10	int: 56.87	Xuezhikang + routine treatment	Routine treatment	8 weeks	TC, TG, HDL−C, LDL−C, FPG
					cont: 57.37				
Sui, (2003)	Diabetes + hyperlipidaemia	5	48:32	24:24/22:10	int: 45–73	Xuezhikang	Amaranth capsules	8 weeks	TC, TG, HDL−C, FPG, ISI, HbA1c
					cont: 42–70				
Deng et al. (2003)	Diabetes + hyperlipidaemia	6	30:26	34:22	52.6 ± 7.3	Xuezhikang + routine treatment	Routine treatment	weeks	TC, TG, HDL−C, LDL−C
Zeng et al. (2001)	Diabetes + hyperlipidaemia	5	22:24	12:10/12:12	int: 47–71	Xuezhikang + routine treatment	Routine treatment	8 weeks	TC, TG, HDL−C, LDL−C, FPG, HbA1c
					cont: 52–74				
Gentile et al. (2000)	Diabetes + hypercholesterolemia	6	80:86	54:26/63:23	int: 58 ± 5	Lovastatin	Placebo	24 weeks	TC, TG, HDL−C, LDL−C
					cont: 61 ± 6				
Peng et al. (2000)	Diabetes + hyperlipidaemia	5	34:34	22:12/25:9	int: 44.1 ± 9.0	Xuezhikang + routine treatment	Routine treatment	24 weeks	TC, TG, FPG, HbA1c
					cont: 46.7 ± 9.6				
Chang et al. (1998)	Hypertension + hyperlipidaemia	6	32:30	30:32	56.7 ± 6.2	Lovastatin	Inositol nicotinate	2 months	TC, TG, HDL−C
Wang et al. (1997)	Diabetes + hyperlipidaemia	5	17:17	9:25	60 ± 4	Xuezhikang	Gemfibrozil	4 weeks	TC, TG, HDL−C, ISI

Note:*Daixiefang: a prescription of traditional Chinese medicine is used to improve the metabolism, including Prunus persica (L.) batsch, Rheum officinale Baill., Alisma plantago−aquatica Linn., trichosanthis radix, Dendranthema indicum(L) Des Moul., and Crataegus pinnatifida, Sargassum.

### Risk of Bias

The median score on the PEDro (on a scale of 0–10) for the included trials was 6. Among the 30 original trials, ten (33.3%) trials were considered “fair” quality and had a medium risk of bias (PEDro score ranging from 4 to 5). Seventeen (56.7%) trials were considered “good” quality and had a low risk of bias (PEDro score ranging from 6 to 8). Three (10.0%) trials were considered “excellent” quality and had a low risk of bias (PEDro score of 9) (Table 1). The main reasons for the risk of bias were nonblinded therapists (24 trials [80.0%]), nonblinded assessors (30 trials [100.0%]), not performing concealed allocation (27 trials [90.0%]), and withdrawal rates higher than 15% (8 trials [26.7%]) **(** Supplementary Table S2).

### Effects of RYR Preparations on Mortality and MACEs

**FIGURE 2:**
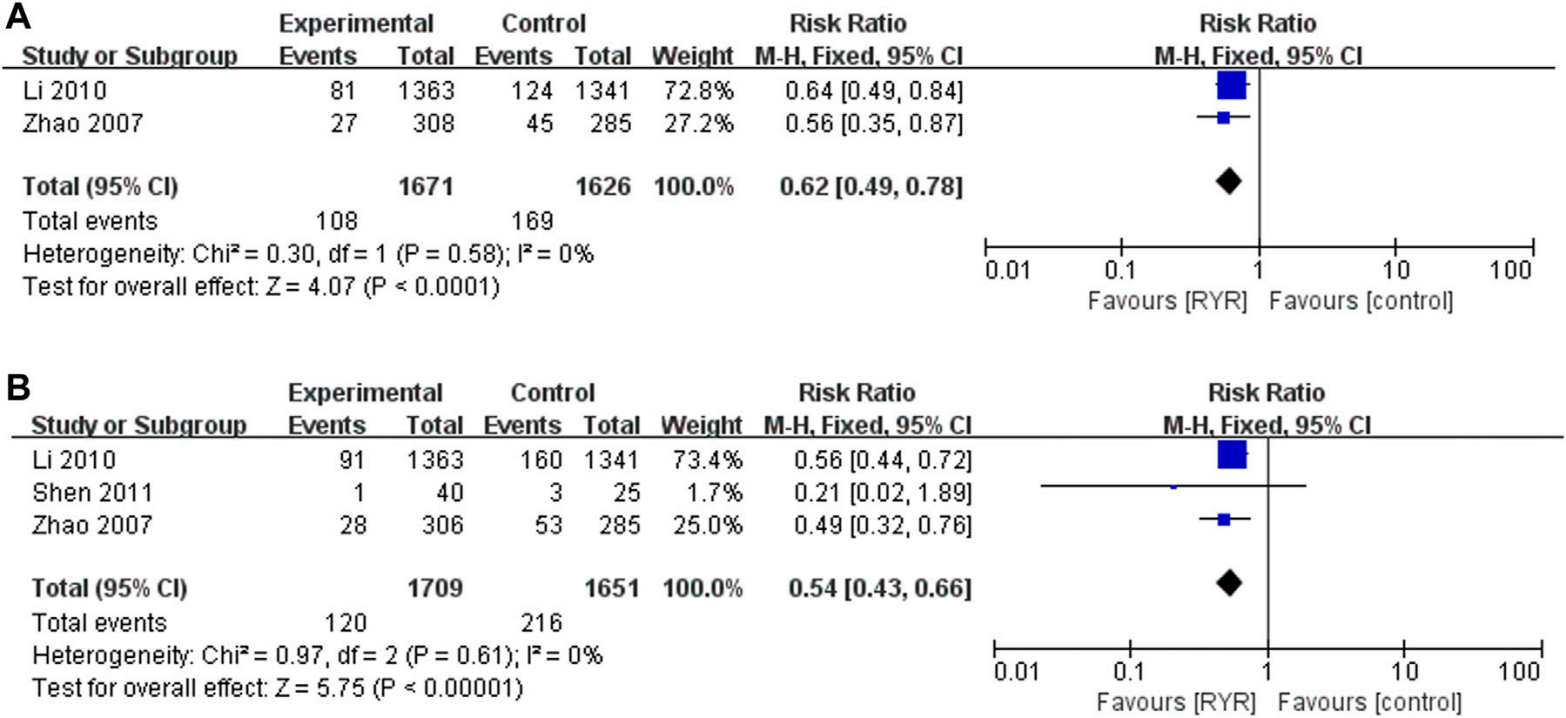
Forest plots of the risk of **(A)** mortality and **(B)** MACEs.

Two trials (Zhao et al., 2007; Li et al., 2010) including 3297 participants reported therapeutic effects of RYR on mortality, and three trials (Zhao et al., 2007; Li et al., 2010; Shen, 2011) including 3360 participants reported its therapeutic effects on MACEs. RYR preparations significantly reduced the mortality (RR = 0.62, 95% CI [0.49, 0.78]), and MACEs (RR = 0.54, 95% CI [0.43, 0.66]) compared with control group. Furthermore, there was no heterogeneity among the studies (Figure 2).

### Effects of RYR Preparations on MetS Risk Factors

#### Blood Glucose Parameters (FPG, HbA1c, HOMA-IR, and ISI)

**FIGURE 3:**
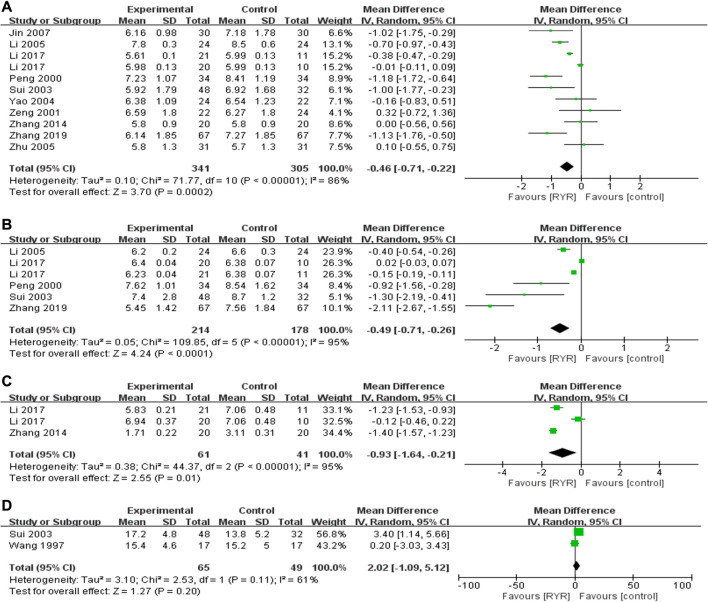
Forest plots of **(A)** FPG, **(B)** HbA1c, **(C)** HOMA−IR, and **(D)** ISI.

Ten trials including 646 participants reported therapeutic effects of RYR on FPG, and RYR preparations significantly reduced FPG level (−0.46 mmol/L, 95% CI [−0.71, −0.22]) compared with control group **(** Figure 3A). There was high heterogeneity among the studies of FPG and subgroup analysis was performed. RYR preparations significantly reduced FPG level (−0.92 mmol/L, 95% CI [−1.26, −0.58], *p <* 0.00001, *I* ^2^ = 41%) in 40- to 50-year-old patients with more notable effects and low heterogeneity, while other subgroups had high heterogeneity. Besides, compared with conventional therapy, RYR plus conventional therapy reduced FPG level (−0.53 mmol/L, 95% CI [−0.93, −0.12], *p =* 0.01, *I* ^2^ = 67%) with medium heterogeneity, while other subgroups had high heterogeneity **(** Supplementary Figure S1 **)**. Five trials including 392 participants reported its therapeutic effects on HbA1c, and RYR preparations reduced HbA1c level (−0.49, 95% CI [−0.71, −0.26]) compared with control group **(** Figure 3B). There was high heterogeneity among the studies of HbA1c, hence subgroup analysis was performed. However, compared with conventional therapy, RYR plus conventional therapy reduced HbA1c level (−1.48 mmol/L, 95% CI [−2.13, −0.84], *p <* 0.00001, *I* ^2^ = 94%) with high heterogeneity, and the heterogeneity remains unexplained in other subgroups **(** Supplementary Figure S2 **)**. Two trials including 102 participants reported therapeutic effects on HOMA−IR, and two trials including 114 participants reported therapeutic effects on ISI. RYR preparations lowered HOMA−IR (−0.93, 95% CI [−1.64, −0.21]) while did not increase ISI (2.02, 95% CI [−1.09, 5.12]) compared with control group **(** Figures 3C,D−). There was high heterogeneity among the studies of HOMA-IR and ISI, yet subgroup analysis could not be performed and the heterogeneity could not be fully investigated due to the limited number of studies and the small sample sizes. Therefore, further validation on existing results is required.

#### Lipid Profile Parameters (TC, TG, LDL-C, and HDL-C)

**FIGURE 4:**
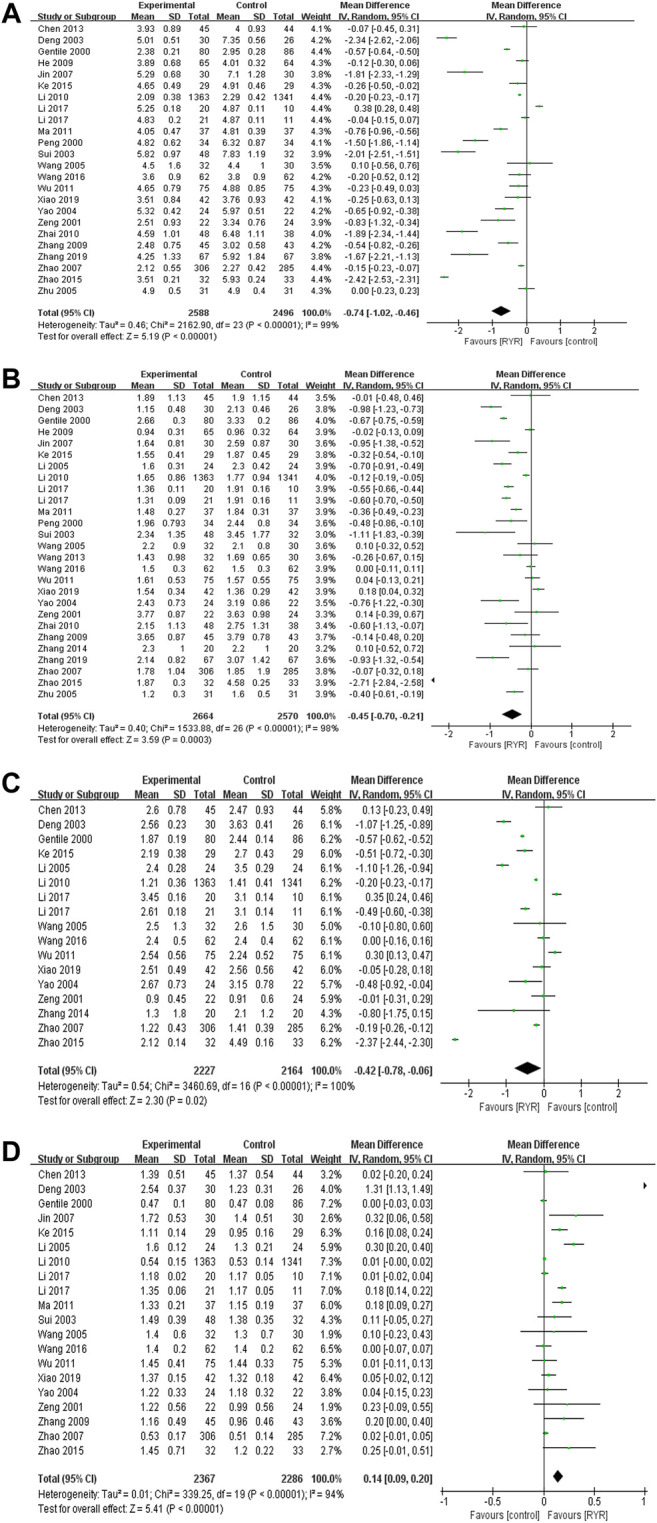
Forest plots of **(A)** TC, **(B)** TG, **(C)** LDL−C, and **(D)** HDL−C.

Twenty−three trials including 5084 participants reported therapeutic effects of RYR on TC, 26 trials including 5234 participants on TG, 16 trials including 4391 participants on LDL, and 19 trials including 4653 participants on HDL. RYR preparations reduced the levels of TC (−0.74 mmol/L, 95% CI [−1.02, −0.46]), TG (−0.45 mmol/L, 95% CI [−0.70, −0.21]) and LDL−C (−0.42 mmol/L, 95% CI [−0.78, −0.06]), while increased HDL−C level (0.14 mmol/L, 95% CI [0.09, 0.20]) compared with control group **(** Figure 4). There was high heterogeneity among the studies of TC, TG, LDL−C and HDL−C, hence subgroup analysis was performed. RYR preparations significantly reduced the levels of TC (−1.55 mmol/L, 95% CI [−1.85, −1.25]*, p* < 0.00001*, I* ^2^ = 0%) and TG (−0.70 mmol/L, 95% CI [−0.90, −0.50], *p* < 0.00001*, I* ^2^ = 24%) in 40− to 50−year−old patients with more notable effects and low heterogeneity, while other subgroups had high heterogeneity. It also significantly reduced TG level (−0.59 mmol/L, 95% CI [−0.66, −0.52], *p* < 0.00001*, I* ^2^ = 0%) in a higher proportion of women (male: female = 1:1.5) with more notable effects and low heterogeneity, while other subgroups had high heterogeneity. Moreover, RYR preparations significantly reduced the levels of TC (−0.19 mmol/L, 95% CI [−0.23, −0.15], *p* < 0.00001*, I* ^2^ = 26%), TG (−0.12 mmol/L, 95% CI [−0.18, −0.05], *p* = 0.0005, *I* ^2^ = 0%), LDL (−0.20 mmol/L, 95% CI [−0.23, −0.17], *p* < 0.00001, *I* ^2^ = 0%), and increased the level of HDL (0.01 mmol/L, 95% CI [0.00, 0.02], *p* = 0.02, *I* ^2^ = 0%) in the intervention duration of >12 months with mild effects and low heterogeneity, while other subgroups had high heterogeneity. However, compared with conventional therapy, RYR plus conventional therapy reduced the levels of TC (−1.48 mmol/L, 95% CI [−2.13, −0.84], *p <* 0.00001, *I* ^2^ = 94%), TG (−0.53 mmol/L, 95% CI [−0.78, −0.28], *p <* 0.0001, *I* ^2^ = 75%) and LDL (−0.70 mmol/L, 95% CI [−1.12, −0.28], *p <* 0.001, *I* ^2^ = 91%), while increased HDL level (0.40 mmol/L, 95% CI [0.03, 0.78], *p =* 0.03, *I* ^2^ = 96%) with high heterogeneity; compared with lipid−lowing drugs, RYR preparations increased HDL level (0.07 mmol/L, 95% CI [0.01, 0.12], *p =* 0.01, *I* ^2^ = 67%) with medium heterogeneity; compared with placebo, RYR preparations reduced the levels TC (−0.83 mmol/L, 95% CI [−1.58, −0.09], *p =* 0.03, *I* ^2^ = 100%) and LDL (−0.83 mmol/L, 95% CI [−1.64, −0.03], *p =* 0.04, *I* ^2^ = 100%) with high heterogeneity **(** Supplementary Figures S3–S6 **)**. In addition, meta−regression analysis revealed that age was a significant moderator of TC (*p* = 0.017) **(** Supplementary Table S3 **)**.

#### Blood Pressure Parameters (MAP, SBP, and DBP)

**FIGURE 5:**
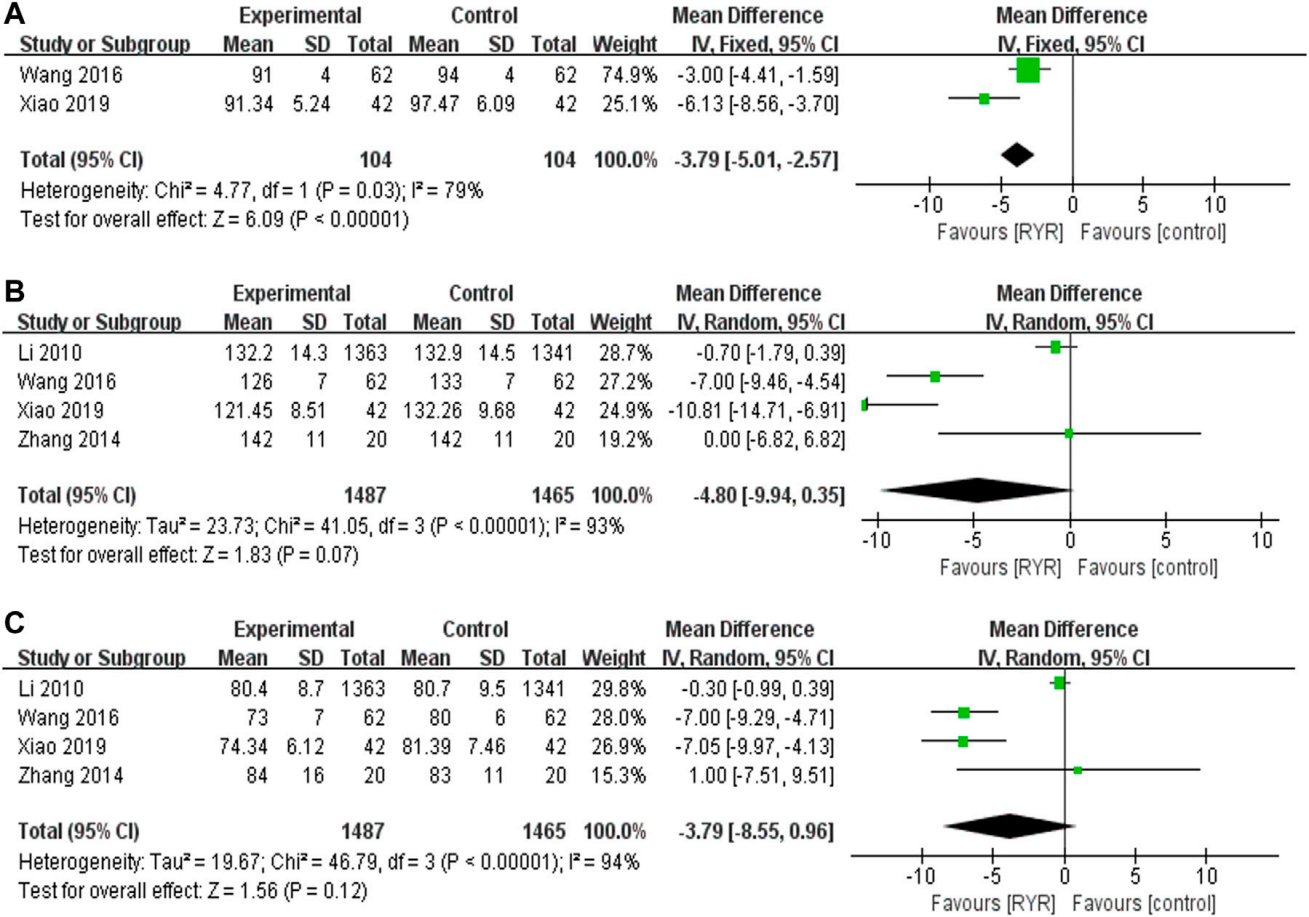
Forest plots of **(A)** MAP, **(B)** SBP, and **(C)** DBP.

Two trials (Wang and Wang, 2016; Xiao 2019) including 208 participants reported therapeutic effects of RYR on MAP, and RYR preparations reduced MAP level (−3.79 mmHg, 95% CI [−5.01, −2.57]) compared with control group **(** Figure 5A). There was high heterogeneity among the studies of MAP, while subgroup analysis could not be performed and the heterogeneity could not be fully investigated due to the limited number of studies and the small sample sizes. Therefore, further validation on existing results is required. Four trials including 2952 participants reported its therapeutic effects on SBP and DBP, while RYR preparations did not reduce SBP or DBP levels compared with control group **(** Figures 5B,C−). There was high heterogeneity among the studies of SBP and DBP and subgroup analysis was performed. RYR preparations significantly reduced SBP level (−8.59 mmHg, 95% CI [−12.28, −4.91], *p <* 0.00001, *I* ^2^ = 62%) and DBP level (−7.02 mmHg, 95% CI [−8.82, −5.22], *p <* 0.00001, *I* ^2^ = 0%) in 60 to 70-year-old patients with more notable effects and low to medium heterogeneity, while other subgroups had high heterogeneity. RYR preparations significantly reduced SBP level (−6.72 mmHg, 95% CI [−11.31, −2.14], *p =* 0.004, *I* ^2^ = 74%) and DBP level (−6.36 mmHg, 95% CI [−8.93, −3.79], *p <* 0.00001, *I* ^2^ = 39%) in the intervention duration of ≤3 months with more notable effects and low to medium heterogeneity, while other subgroups had high heterogeneity. Furthermore, compared with lipid−lowing drugs, RYR preparations significantly reduced SBP level (−8.59 mmHg, 95% CI [−12.28, −4.91], *p <* 0.00001, *I* ^2^ = 62%) and DBP level (−7.02 mmHg, 95% CI [−8.82, −5.22], *p <* 0.00001, *I* ^2^ = 0%) with more notable effects and low to medium heterogeneity, while other subgroups had high heterogeneity **(** Supplementary Figures S7–S8 **)**.

### Effects of RYR Preparations on Adverse Reactions

**FIGURE 6:**
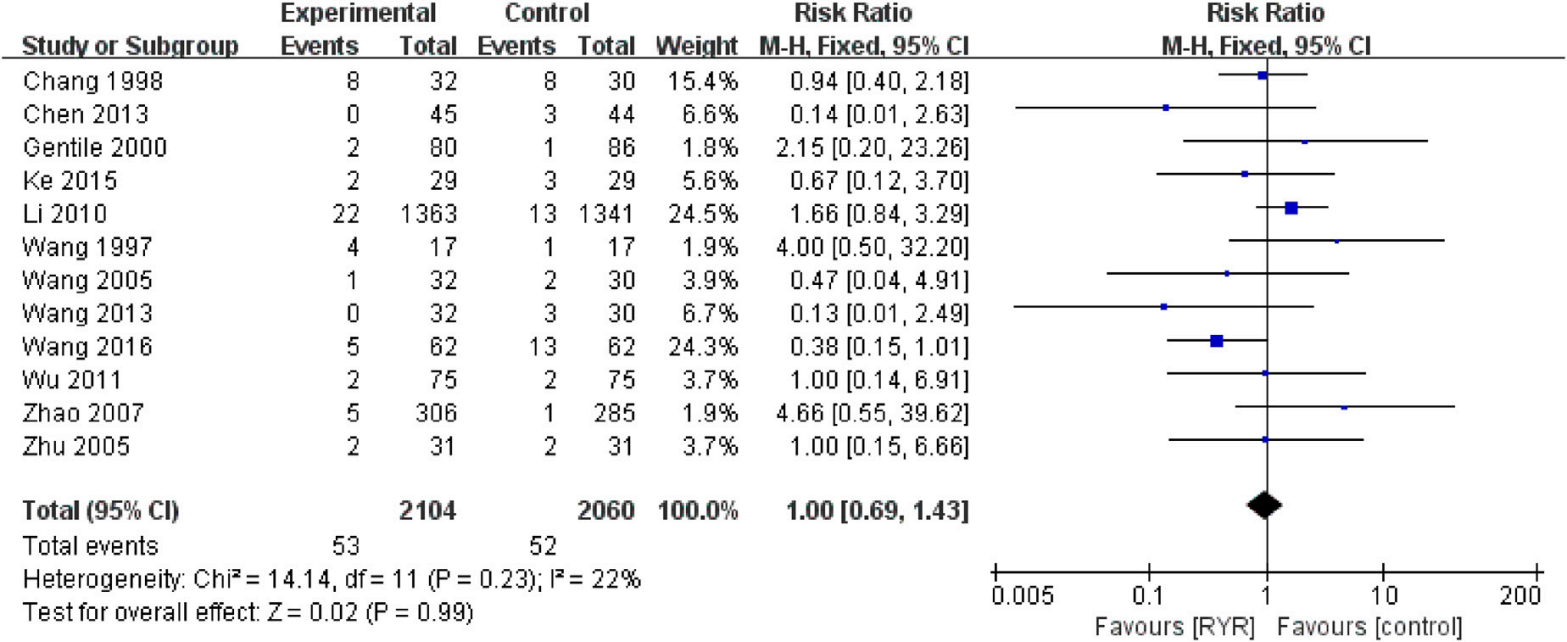
Forest plot of the adverse reactions.

Twelve trials including 4164 participants reported adverse reactions, including gastrointestinal disorders (nausea, abdominal pain, diarrhoea, flatulence, etc.), mental−neurological symptoms (depression, etc.), oedema, myalgia, dizziness, and allergic reactions. RYR preparations did not increase the incidence of adverse reactions compared with control group (1.00, 95% CI [0.69, 1.43]) and there was low heterogeneity (*I* ^2^ = 22%) among the studies **(** Figure 6).

### Sensitivity Analysis

Sensitivity analysis demonstrated the pooled effect estimates of mortality, MACEs, FPG, HbA1c, TC, TG, LDL-C, HDL-C, MAP, and adverse reactions did not substantially modify, and these results are robust. However, the pooled effect estimates showed no significant differences for HOMA−IR after excluding the study by Zhang and Wu, 2014, which indicated that this result was not robust. The reason might be related to low methodological quality of Zhang and Wu, 2014 (PEDro score = 4) with no concealed allocation and blinding, and the high risk of selection bias might exaggerate the effect of RYR preparations in lowering HOMA−IR. Furthermore, the pooled effect estimates showed significant differences in SBP and DBP after excluding the study by Li et al., 2010a, which also indicated that this result was not robust. The reason might be related to long duration of treatment in Li et al., 2010b (4.5 years), and the effects of RYR preparations on SBP and DBP might not be significant in the long follow−up time.

### Publication Bias

**FIGURE 7:**
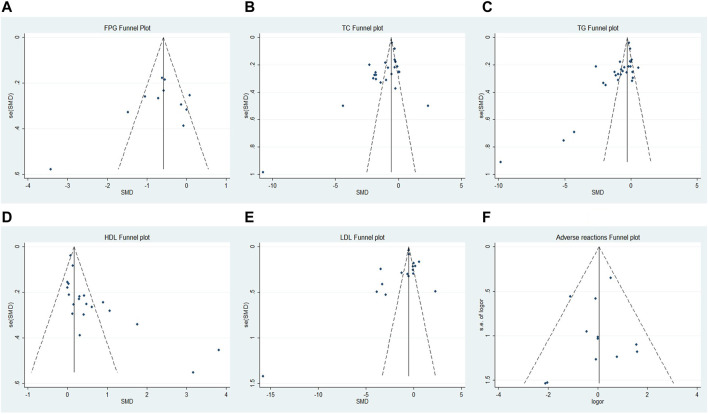
Funnel plots of **(A)** FPG, **(B)** TC, **(C)** TG, **(D)** HDL−C, **(E)** LDL−C, and **(F)** adverse reactions.

Both visual inspection of the funnel plot and Egger’s test demonstrated no evidence of publication bias in FPG (*p* = 0.265), LDL-C (*p* = 0.241) and adverse reactions (*p* = 0.573). However, there was publication bias for TC (*p* = 0.040), TG (*p* = 0.001) and HDL−C (*p* = 0.002) (Figure 7 and Supplementary Table S4).

### Quality of the Evidence

All of the outcomes of interest were assessed with GRADE and evidence profiles are shown in Table 2. There was high-quality evidence on mortality, MACEs and adverse reactions; medium−quality evidence on FPG, HbA1c, HOMA-IR, TC, LDL-C, MAP and DBP; low-quality evidence on TG, HDL-C and SBP; and very low−quality evidence on ISI. The certainty of the evidence ranged from very low to high, which might be attributed to the risk of bias, reporting bias, inconsistency, and imprecision, indicating that these estimates are uncertain and that future studies are likely to influence our confidence in the results.

**TABLE 2:** GRADE quality assessment.

		Bias	Inconsistency	Indirectness	Imprecision	Publication bias	Rating
Mortality, 2 RCTs (*n* = 3297)	RR 0.62, 95%CI 0.49 to 0.78, I^2^ 0%	0	0	0	0	0^d^	4: high
MACEs, 3 RCTs (*n* = 3360)	RR 0.54, 95%CI 0.43 to 0.66, I^2^ 0%	0	0	0	0	0^d^	4: high
FPG, 10 RCTs (*n* = 646)	MD −0.46, 95% CI −0.71to −0.22, I^2^ 86%	0	−1^b^	0	0	0	3: medium
HbAc1, 5 RCTs (*n* = 392)	MD −0.49, 95%CI −0.71 to −0.26, I^2^ 95%	0	−1^b^	0	0	0^d^	3: medium
HOMA−IR, 2 RCTs (*n* = 102)	MD −0.93, 95%CI −1.64 to −0.21, I^2^ 95%	0	−1^b^	0	0	0^d^	3: medium
ISI, 2 RCTs (*n* = 114)	MD 2.02, 95%CI −1.09 to 5.12, I^2^ 61%	−1^a^	−1^b^	0	−1^c^	0^d^	1: very low
TC, 23 RCTs (*n* = 5114)	MD −0.74, 95%CI −1.02 to −0.46, I^2^ 99%	0	0	0	0	−1^e^	3: medium
TG, 26 RCTs (*n* = 5264)	MD −0.45, 95%CI −0.70 to −0.21, I^2^ 98%	0	−1^b^	0	0	−1^e^	2: low
LDL, 16 RCTs (*n* = 4421)	MD −0.42, 95%CI −0.78 to −0.06, I^2^ 100%	0	−1^b^	0	0	0	3: medium
HDL, 19 RCTs (*n* = 4683)	MD 0.14, 95%CI 0.09 to 0.20, I^2^ 94%	0	−1^b^	0	0	−1^e^	2: low
MAP, 2 RCTs (*n* = 208)	MD −3.79, 95%CI −5.01 to −2.57, I^2^ 79%	0	−1^b^	0	0	0^d^	3: medium
SBP, 4 RCTs (*n* = 2952)	MD −4.80, 95%CI −9.94 to 0.35, I^2^ 93%	0	−1^b^	0	−1^c^	0^d^	2: low
DBP, 4 RCTs (*n* = 2952)	MD −3.79, 95%CI −8.55 to 0.96, I^2^ 94%	0	0	0	−1^c^	0^d^	3: medium
Adverse reactions, 12 RCTs (*n* = 4194)	RR 1.00, 95%CI 0.69 to 1.43, I^2^ 22%	0	0	0	0	0	4: high

a Downgraded one place due to the majority of trials scoring <6 on the PEDro scale.

b Downgraded one place due to unexplained heterogeneity.

c Downgraded one place due to the small sample size or the combined effect size passing the invalid line.

d Funnel plots not completed due to <10 studies in the meta−analysis.

e Downgraded one place due to publication bias.

## Discussion

### Summary of Main Results

This systematic review and meta-analysis showed robust and consistent findings that RYR preparations reduce mortality, MACEs, and risk factors of MetS. Therefore, RYR preparations might be an effective treatment to improve health outcomes and might assist in decreasing the MetS risk factors and preventing progression to CVD. Taken together, the results of these studies support the beneficial effects of RYR preparations across different clinical settings.

### Clinical Benefits and Mechanism of Improving MetS Biochemical Parameter

Blood glucose parameters are related to diabetes. A recent nationwide epidemiological data demonstrated that elevated FPG increase the risk of CVD (Kaneko et al., 2021); each 1% higher HbA1c, independent of diabetes status, is associated with an 18% greater risk of myocardial infarction (de Jong et al., 2020); HOMA−IR is positive correlation with CVD risk (Lu et al., 2020). This meta−analysis observed that RYR preparations significantly reduced the levels of FPG, HbA1c and HOMA−IR, indicating its promising application in improving diabetes and CVD. Previous studies indicated that RYR could stimulate muscarinic M3 receptors in pancreatic cells and augment insulin release to lower plasma glucose (Chen and Liu, 2006); and inhibit high glucose−induced proangiogenic cells senescence and oxidative stress, thereby decreasing the vascular complications of diabetes (Liu et al., 2017), which might partly account for the improved blood glucose parameters after RYR preparations treatment.

Blood lipid profiles play a critical role in arteriosclerotic CVD. A cross−sectional study in T2 diabetes found that a lower TG was associated with a reduced CVD risk in the short term (Ren et al., 2018); Mortality and morbidity from CVD were reduced via primary and secondary prevention through lipid−lowering therapy targeting LDL−C (Collins et al., 2016, Serban et al., 2016); HDL has antidiabetic functions by increasing insulin sensitivity and β−cell function (Cochran et al., 2021). As the results of the present study, the reductions of TC, TG, LDL−C and the increase of HDL−C are likely to be clinically significant in improving glucose and lipid metabolism and then reducing CAD risk. Previous studies indicated that RYR could lower lipid by inhibiting HMG−CoA reductase (Banach et al., 2018), which might partly account for the improved blood lipid profiles after RYR preparations treatment.

High blood pressure can result in heart failure, heart attack, stroke, and kidney disease (Desai, 2020), and MAP is a predictor of all−cause and CVD mortality in the middle−aged and elderly populations (Sun et al., 2020). In the present study, RYR preparations significantly reduced MAP level of 3.79 mmHg, it also significantly reduced SBP and DBP level in 60−70 years old patients and in the intervention duration of ≤3 months. In addition, previous studies indicated that RYR could improve artery stiffness, endothelial dysfunction and inflammation by promoting and stabilizing the expression of endothelial nitric oxide synthase (eNOS) (Hu et al., 2020; Cicero et al., 2021). Therefore, the mechanism of RYR on lowering blood pressure might be due to improving endothelial dysfunction and inflammation.

### Consistency and Disagreement With Other Studies

Up to now, used as assistance for CVD prevention, nutraceuticals has mainly been focusing on lipid-lowering, as referred in the documents produced by the International Lipid Expert Panel (ILEP). As lipid-lowering nutraceuticals, RYR plays an important role in the reduction of inflammation-related residual CVD risk (Ruscica et al., 2021). Recent studies suggested that RYR could be used in patients at cardiovascular risk who had not reached the LDL-C target, but not eligible for statins or unable to tolerate statins (Banach et al., 2019). RYR has been validated to decrease blood sugar, lower cholesterol, control high blood pressure, and inhibit inflammation (Patel, 2016). Therefore, these findings indicated that RYR could treat MetS and improve the prognosis of CVD, which is consistent with our findings. Recent reports identified a low prevalence of suspected adverse effects associated with RYR (Banach et al., 2021), confirmed its safety in hypercholesterolemic patients, even in patients with statin intolerance (Fogacci et al., 2019; Cicero et al., 2021), and our findings also supported the safety of RYR.

Previous study indicated that the lipid−lowering effect of RYR was not influenced by age or gender in physicians (Verhoeven et al., 2013). However, in the subgroup analysis of the present study, RYR preparations significantly reduced the levels of FPG, TC and TG in 40−50 years old patients, SBP and DBP in 60−70 years old patients, and TG in women with more notable effects and low to medium heterogeneity, which suggested that RYR preparations might have better therapeutic advantages in these populations. The different results on age and gender might be related to the basal metabolic rate, vascular dysfunction, and sex differences in TG metabolism: previous studies indicated that basal metabolic rate was significantly higher in middle age (40−50 years old) compared with old age (60−70 years old) (St−Onge and Gallagher, 2010); blood pressure has an increasingly positive association with vascular dysfunction and vascular stiffness as age increases (Wen et al., 2015); women have better clearance of meal−related TG and estrogen is acting directly in the liver to reduce TG level (Palmisano et al., 2018). Therefore, RYR preparations might have better effects on blood glucose and blood lipid in middle−aged people by regulating basal metabolic rate, have better effect on SBP and DBP in old−aged people by improving vascular dysfunction and artery stiffness, and have better effect on TG in women by virtue of better TG clearance. Previous clinical trials showed that MAP, SBP, or DBP were not significantly different between Xuezhikang and placebo at 6, 12, 24, and 48 months (Li et al., 2010). While the present study found that RYR preparations could reduce MAP level, it also reduced SBP and DBP levels in the intervention duration of ≤3 months, which indicated that short duration of RYR preparations intervention might be beneficial to reducing blood pressure. Previous studies indicated that nutraceuticals (berberine, RYR, policosanol) significantly reduced the levels of TC, LDL and HOMA−IR after a 12-months treatment (Marazzi et al., 2011). Nevertheless, in the subgroup analysis of the present study, RYR preparations had a small but significant improvement on TC, TG, LDL and HDL in the intervention duration of >12 months with low heterogeneity, which provided more evidence on long−term use of RYR preparations on reducing blood lipid. Previous systematic review showed that RYR plus conventional therapy lowered the levels of TC, LDL, and SBP compared with conventional therapy, and the levels of TC and LDL compared with placebo plus conventional therapy, while RYR plus statins lowered the levels of TC, TG, LDL, SBP, and DBP compared with statins (Xiong et al., 2017). However, the *I* ^2^ ranged from 47 to 98%, suggesting the medium to high heterogeneity among the included studies. In the present study, RYR preparations reduced the levels of SBP and DBP, while increased HDL level compared with lipid−lowing drugs (*I* ^2^ ranged from 0 to −95%); RYR preparations plus conventional therapy reduced the levels of FPG, HbA1c, TC, TG and LDL, while increased HDL level compared with conventional therapy (*I* ^*2*^ ranged from 58 to −96%); RYR preparations reduced TC and LDL levels compared with placebo (*I* ^2^ = 100%), which was partly consistent with the previous study. Additionally, the present study further indicated that RYR preparations showed positive effect on DBP with low heterogeneity (I^2^ = 0%), and it also beneficially regulate FPG, HbA1c and HDL levels, which have not been reported in the systematic review of Xiong et al., 2017.

### Strengths and Limitations

There have been many previous meta-analyses and reviews about RYR preparations, while these analyses mainly focused on lipid profiles and safety (Dujovne, 2017; Banach et al., 2019). The present meta-analysis mainly focused on the efficacy and safety of RYR preparations on MetS, showed the association between biochemical parameters and clinical benefits, all of which might provide the evidence base for clinical practice. Moreover, the results showed promising effects of RYR preparations on improving glucose and lipid metabolism, modifying cardiovascular risk, effectively reducing the occurrence of mortality and MACEs, which have not been evaluated in the previous meta−analysis. Furthermore, the results demonstrated that RYR preparations were well tolerated with a favourable safety profile.

There are some limitations of our study. First, most of the studies included were of low and moderate methodological quality, which might increase the risk of bias, and therefore, only low- and medium-level evidence can be established. Second, most of the included studies focused on the Chinese population, except for one study conducted in Italy, thus, it is not clear whether the effect of RYR preparations could be extended to other populations. Third, although some of the outcomes in subgroup analysis showed more notable effects and low heterogeneity, sample sizes of these studies were small, thus the efficacy needs further investigation. Finally, regarding the high heterogeneity, different patient ages and sexes, different intervention durations and intervention types were taken into account; however, although subgroup analysis and meta−regression analyses were conducted, the heterogeneities were not eliminated for HbA1c, HOMA−IR, ISI and MAP, and differences remained among the studies in terms of the limited number of studies, sample sizes, race, religious beliefs, and concern regarding the disease; thus, these results should be interpreted with caution. Therefore, more large−sample and high−quality RCTs in broader populations are needed to provide additional data support for the efficacy of RYR on MetS.

## Conclusion

The evidence of the current review suggests that RYR preparations significantly reduce the occurrence of mortality and MACEs in MetS and improve blood glucose, lipid profiles, and blood pressure. Taken over the long term, RYR preparations could improve clinical endpoints, prevent metabolic diseases, and reduce the risk of CAD. However, due to the low− and moderate−quality evidence of the included studies and the heterogeneity among the different studies, the results of our study should be evaluated with prudence, and more high−quality clinical studies are required to provide stronger evidence for RYR in the treatment of MetS. These findings suggest that RYR preparations should be taken into consideration for the prevention and treatment of MetS due to its favourable effects on multiple risk factors for hyperglycaemia, dyslipidaemia, and hypertension and its acceptable safety profile.

## Data Availability

The original contributions presented in the study are included in the article/Supplementary Material, further inquiries can be directed to the corresponding authors.
