# Supplementary material for: Red Yeast Rice Preparations Reduce Mortality, Major Cardiovascular Adverse Events, and Risk Factors for Metabolic Syndrome: A Systematic Review and Meta−analysis
Source: Front Pharmacol. 2022 Feb 21;13:744928. doi: 10.3389/fphar.2022.744928 (PMC8899821; doi:10.3389/fphar.2022.744928)
Supplement: Supplementary file 1 [file DataSheet3.docx]

Supplementary Table S1 The details of Chinese patent medicines of the included trials

| Study | Formulation | Source | Species | Quality control reported? (Y/N) | Chemical analysis reported?(Y/N) |
| --- | --- | --- | --- | --- | --- |
| Zhang 2019 | Lovastatin Tablet, 20 mg | Chaoyang Fuxiang Pharmaceutical Co., Ltd. | Lovastatin | Y-Prepared according to Chinese pharmacopeia(2015) | Y-HPLC |
| Xiao 2019 | Xuezhikang capsule | Beijing Peking University WBL Biotech Co., Ltd. | Red yeast rice | Y-Prepared according to Chinese pharmacopeia(2015) | Y- HPLC |
| Li 2017 | Xuezhikang Tablet | Beijing Peking University WBL Biotech Co., Ltd. | Red yeast rice | Y-Prepared according to Chinese pharmacopeia(2015) | Y- HPLC |
| Wang 2016 | Xuezhikang capsule | Beijing Peking University WBL Biotech Co., Ltd. | Red yeast rice | Y-Prepared according to Chinese pharmacopeia(2015) | Y- HPLC |
| Zhao 2015 | Xuezhikang capsule | Beijing Peking University WBL Biotech Co., Ltd. | Red yeast rice | Y-Prepared according to Chinese pharmacopeia(2015) | Y- HPLC |
| Ke 2015 | Xuezhikang capsule | Beijing Peking University WBL Biotech Co., Ltd. | Red yeast rice | Y-Prepared according to Chinese pharmacopeia(2015) | Y- HPLC |
| Zhang 2014 | Xuezhikang capsule | Beijing Peking University WBL Biotech Co., Ltd. | Red yeast rice | Y-Prepared according to Chinese pharmacopeia(2010) | Y- HPLC |
| Chen 2013 | Xuezhikang capsule | Beijing Peking University WBL Biotech Co., Ltd. | Red yeast rice | Y-Prepared according to Chinese pharmacopeia(2010) | Y- HPLC |
| Wang 2013 | Xuezhikang capsule | Beijing Peking University WBL Biotech Co., Ltd. | Red yeast rice | Y-Prepared according to Chinese pharmacopeia(2010) | Y- HPLC |
| Wu 2011 | Xuezhikang capsule | Beijing Peking University WBL Biotech Co., Ltd. | Red yeast rice | Y-Prepared according to Chinese pharmacopeia(2010) | Y- HPLC |
| Ma 2011 | Xuezhikang capsule | Beijing Peking University WBL Biotech Co., Ltd. | Red yeast rice | Y-Prepared according to Chinese pharmacopeia(2010) | Y- HPLC |
| Shen 2011 | Xuezhikang capsule | Beijing Peking University WBL Biotech Co., Ltd. | Red yeast rice | Y-Prepared according to Chinese pharmacopeia(2010) | Y- HPLC |
| Li 2010 | Xuezhikang capsule | Beijing Peking University WBL Biotech Co., Ltd. | Red yeast rice | Y-Prepared according to Chinese pharmacopeia(2010) | Y- HPLC |
| Zhai 2010 | Xuezhikang capsule | Beijing Peking University WBL Biotech Co., Ltd. | Red yeast rice | Y-Prepared according to Chinese pharmacopeia(2010) | Y- HPLC |
| Li 2009 | Xuezhikang capsule | Beijing Peking University WBL Biotech Co., Ltd. | Red yeast rice | Y-Prepared according to Chinese pharmacopeia(2005) | Y- HPLC |
| He 2009 | Xuezhikang capsule | Beijing Peking University WBL Biotech Co., Ltd. | Red yeast rice | Y-Prepared according to Chinese pharmacopeia(2005) | Y- HPLC |
| Zhang 2009 | Xuezhikang capsule | Beijing Peking University WBL Biotech Co., Ltd. | Red yeast rice | Y-Prepared according to Chinese pharmacopeia(2005) | Y- HPLC |
| Jin 2007 | Xuezhikang capsule | Beijing Peking University WBL Biotech Co., Ltd. | Red yeast rice | Y-Prepared according to Chinese pharmacopeia(2005) | Y- HPLC |
| Zhao 2007 | Xuezhikang capsule | Beijing Peking University WBL Biotech Co., Ltd. | Red yeast rice | Y-Prepared according to Chinese pharmacopeia(2005) | Y- HPLC |
| Wang 2005 | Xuezhikang capsule | Beijing Peking University WBL Biotech Co., Ltd. | Red yeast rice | Y-Prepared according to Chinese pharmacopeia(2005) | Y- HPLC |
| Zhu 2005 | Xuezhikang capsule | Beijing Peking University WBL Biotech Co., Ltd. | Red yeast rice | Y-Prepared according to Chinese pharmacopeia(2005) | Y- HPLC |
| Li 2005 | Xuezhikang capsule | Beijing Peking University WBL Biotech Co., Ltd. | Red yeast rice | Y-Prepared according to Chinese pharmacopeia(2005) | Y- HPLC |
| Yao 2004 | Xuezhikang capsule | Beijing Peking University WBL Biotech Co., Ltd. | Red yeast rice | Y-Prepared according to Chinese pharmacopeia(2000) | Y- HPLC |
| Sui 2003 | Xuezhikang capsule | Beijing Peking University WBL Biotech Co., Ltd. | Red yeast rice | Y-Prepared according to Chinese pharmacopeia(2000) | Y- HPLC |
| Deng 2003 | Xuezhikang capsule | Beijing Peking University WBL Biotech Co., Ltd. | Red yeast rice | Y-Prepared according to Chinese pharmacopeia(2000) | Y- HPLC |
| Zeng 2001 | Xuezhikang capsule | Beijing Peking University WBL Biotech Co., Ltd. | Red yeast rice | Y-Prepared according to Chinese pharmacopeia(2000) | Y- HPLC |
| Gentile 2000 | Lovastatin Tablet, 20 mg | [Commercial Supplier, Ltd.] | Lovastatin | NO | NO |
| Peng 2000 | Xuezhikang capsule | Beijing Peking University WBL Biotech Co., Ltd. | Red yeast rice | Y-Prepared according to Chinese pharmacopeia(2000) | Y- HPLC |
| Chang 1998 | Lovastatin Tablet, 20 mg | [Commercial Supplier, Ltd.] | Lovastatin | NO | NO |
| Wang 1997 | Xuezhikang capsule | Beijing Peking University WBL Biotech Co., Ltd. | Red yeast rice | Y-Prepared according to Chinese pharmacopeia(1995) | Y- HPLC |

Supplementary Table S2 Quality assessment: PEDro scores of included trials

| Study | Eligibility  Criteria | Random  allocation | Concealed  allocation | Groups  similar at  baseline | Participant  blinding | Therapist  blinding | Assessor  blinding | <15% dropouts | Intention-to-treat  Analysis | Between-group  difference reported | Point estimate and variability reported | Total |
| --- | --- | --- | --- | --- | --- | --- | --- | --- | --- | --- | --- | --- |
| Zhang 2019 | Y | Y | N | Y | N | N | N | Y | Y | Y | Y | 6 |
| Xiao 2019 | Y | Y | N | Y | N | N | N | Y | Y | Y | Y | 6 |
| Li 2017 | Y | Y | Y | Y | Y | Y | N | Y | Y | Y | Y | 9 |
| Wang 2016 | Y | Y | N | Y | N | N | N | Y | Y | Y | Y | 6 |
| Zhao 2015 | Y | Y | N | Y | N | N | N | N | Y | Y | Y | 5 |
| Ke 2015 | Y | Y | N | Y | N | N | N | Y | Y | Y | Y | 6 |
| Zhang 2014 | Y | Y | N | Y | N | N | N | N | Y | N | Y | 4 |
| Chen 2013 | Y | Y | N | N | Y | Y | N | Y | Y | Y | Y | 7 |
| Wang 2013 | N | Y | N | Y | N | N | N | Y | Y | Y | Y | 6 |
| Wu 2011 | Y | Y | N | Y | N | N | N | N | Y | Y | Y | 5 |
| Ma 2011 | Y | Y | N | Y | N | N | N | Y | Y | Y | Y | 6 |
| Shen 2011 | Y | Y | N | Y | N | N | N | N | Y | N | Y | 4 |
| Li 2010 | Y | Y | Y | Y | Y | Y | N | Y | Y | Y | Y | 9 |
| Zhai 2010 | Y | Y | N | Y | N | N | N | N | Y | Y | Y | 5 |
| Li 2009 | Y | Y | Y | Y | Y | Y | N | Y | Y | Y | Y | 9 |
| He 2009 | Y | Y | N | Y | N | N | N | Y | Y | Y | Y | 6 |
| Zhang 2009 | Y | Y | N | Y | Y | Y | N | Y | Y | Y | Y | 8 |
| Jin 2007 | N | Y | N | Y | N | N | N | Y | Y | Y | Y | 6 |
| Zhao 2007 | Y | Y | N | Y | Y | Y | N | Y | Y | N | Y | 7 |
| Wang 2005 | Y | Y | N | Y | N | N | N | N | Y | Y | Y | 5 |
| Zhu 2005 | Y | Y | N | Y | N | N | N | Y | Y | Y | Y | 6 |
| Li 2005 | Y | Y | N | Y | N | N | N | Y | Y | Y | Y | 6 |
| Yao 2004 | Y | Y | N | Y | N | N | N | Y | Y | Y | Y | 6 |
| Sui 2003 | N | Y | N | Y | N | N | N | N | Y | Y | Y | 5 |
| Deng 2003 | N | Y | N | Y | N | N | N | Y | Y | Y | Y | 6 |
| Zeng 2001 | Y | Y | N | Y | N | N | N | Y | Y | N | Y | 5 |
| Gentile 2000 | Y | Y | N | Y | N | N | N | Y | Y | Y | Y | 6 |
| Peng 2000 | Y | Y | N | Y | N | N | N | N | Y | Y | Y | 5 |
| Chang 1998 | Y | Y | N | N | Y | N | N | Y | Y | Y | Y | 6 |
| Wang 1997 | Y | Y | N | N | N | N | N | Y | Y | Y | Y | 5 |

Supplementary Table S3 Meta-regression results on heterogeneity

| Outcome | Moderator | *I^2^* | Coefficient | 95% *CI* | *P* |
| --- | --- | --- | --- | --- | --- |
| FPG | Age | 83.45 | 3.33e-16 | [-2.60, 2.60] | 1.000 |
|  | Gender | 83.27 | -0.30 | [-1.81, 1.22] | 0.658 |
|  | Intervention duration | 82.41 | -1.04 | [-3.26, 1.17] | 0.314 |
|  | Intervention type | 75.22 | 0.077 | [-1.36, 1.51] | 0.900 |
| HbA1c | Age | 90.05 | -0.56 | [-4.81, 3.68] | 0.701 |
|  | Gender | 92.98 | -0.56 | [-7.48, 6.35] | 0.759 |
|  | Intervention duration | 87.99 | -1.15 | [-2.62, 0.32] | 0.096 |
|  | Intervention type | 90.01 | -1.28 | [-6.44, 3.87] | 0.396 |
| TC | Age | 94.83 | -3.53 | [-6.35, -0.70] | 0.017 |
|  | Gender | 95.57 | -0.36 | [-4.49, 3.75] | 0.856 |
|  | Intervention duration | 94.91 | -1.32 | [-4.01, 1.36] | 0.318 |
|  | Intervention type | 94.85 | -8.44e-15 | [-5.03, 5.03] | 1.000 |
| TG | Age | 94.59 | 0.10 | [-4.34, 4.54] | 0.963 |
|  | Gender | 94.21 | -0.10 | [-3.02, 2.82] | 0.945 |
|  | Intervention duration | 93.91 | -0.09 | [-3.09, 2.91] | 0.951 |
|  | Intervention type | 94.35 | -0.97 | [-4.86, 2.92] | 0.610 |
| HDL | Age | 85.85 | 1.75 | [-0.37, 3.88] | 0.100 |
|  | Gender | 83.16 | 0.02 | [-1.32, 1.36] | 0.976 |
|  | Intervention duration | 85.99 | 0.10 | [-1.33, 1.52] | 0.886 |
|  | Intervention type | 80.57 | 3.166 | [1.22, 5.11] | 0.003 |
| LDL | Age | 96.71 | -0.52 | [-8.70, 7.65] | 0.893 |
|  | Gender | 97.19 | -0.52 | [-10.07, 9.04] | 0.907 |
|  | Intervention duration | 95.38 | -0.49 | [-6.25, 5.27] | 0.859 |
|  | Intervention type | 96.38 | -2.92 | [-10.24, 4.40] | 0.407 |

Supplementary Table S4 Egger's test

|  | Std_Eff | Coef. | Std. Err. | t | *P*>\|t\| | [95% Conf. Interval] | |
| --- | --- | --- | --- | --- | --- | --- | --- |
| FPG | slope | .1016936 | .6025417 | 0.17 | 0.870 | -1.26135 | 1.464738 |
|  | bias | -2.770772 | 2.328578 | -1.19 | 0.265 | -8.038382 | 2.496837 |
| TC | slope | -.3021163 | .1735564 | -1.74 | 0.096 | -.6620501 | .0578176 |
|  | bias | -2.640586 | 1.208238 | -2.19 | 0.040 | -5.146317 | -.1348543 |
| TG | slope | .1168252 | .1476879 | 0.79 | 0.436 | -.1873437 | .420994 |
|  | bias | -3.56284 | .9988749 | -3.57 | 0.001 | -5.620061 | -1.505619 |
| HDL | slope | -.0870592 | .0962418 | -0.90 | 0.378 | -.2892557 | .1151373 |
|  | bias | 2.600216 | .7256982 | 3.58 | 0.002 | 1.07558 | 4.124851 |
| LDL | slope | -.3201898 | .2312865 | -1.38 | 0.186 | -.8131653 | .1727856 |
|  | bias | -2.169136 | 1.775829 | -1.22 | 0.241 | -5.954227 | 1.615954 |
| Adverse reactions | slope | .2560505 | .488299 | 0.52 | 0.611 | -.8319476 | 1.344049 |
|  | bias | -.4116542 | .7068797 | -0.58 | 0.573 | -1.98668 | 1.163372 |
