# Supplementary material for: Red Yeast Rice Preparations Reduce Mortality, Major Cardiovascular Adverse Events, and Risk Factors for Metabolic Syndrome: A Systematic Review and Meta−analysis
Source: Front Pharmacol. 2022 Feb 21;13:744928. doi: 10.3389/fphar.2022.744928 (PMC8899821; doi:10.3389/fphar.2022.744928)
Supplement: Supplementary file 2 [file DataSheet2.docx]

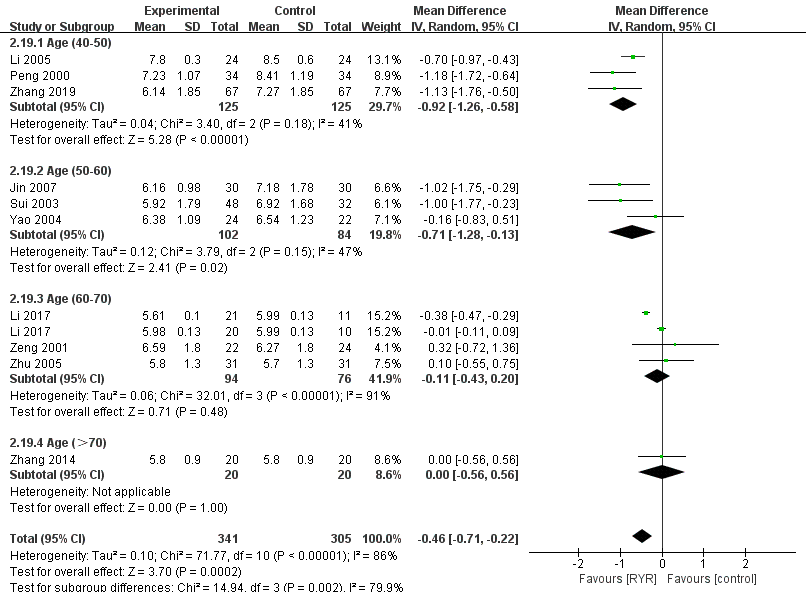

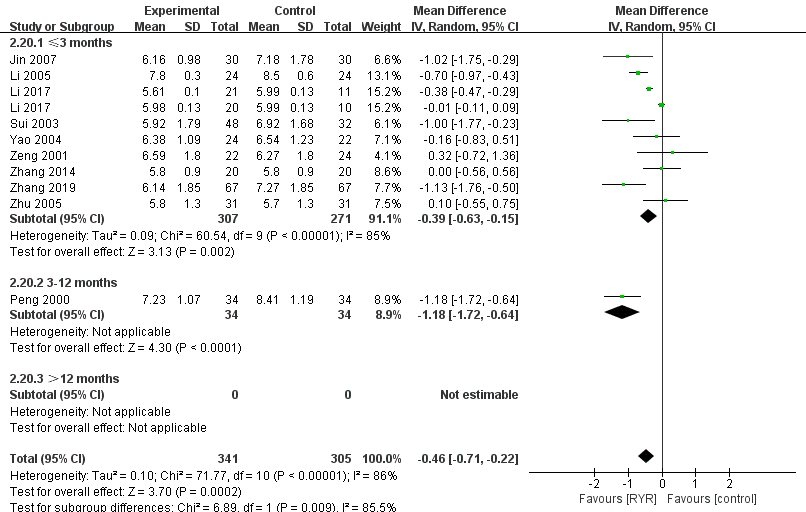


A B


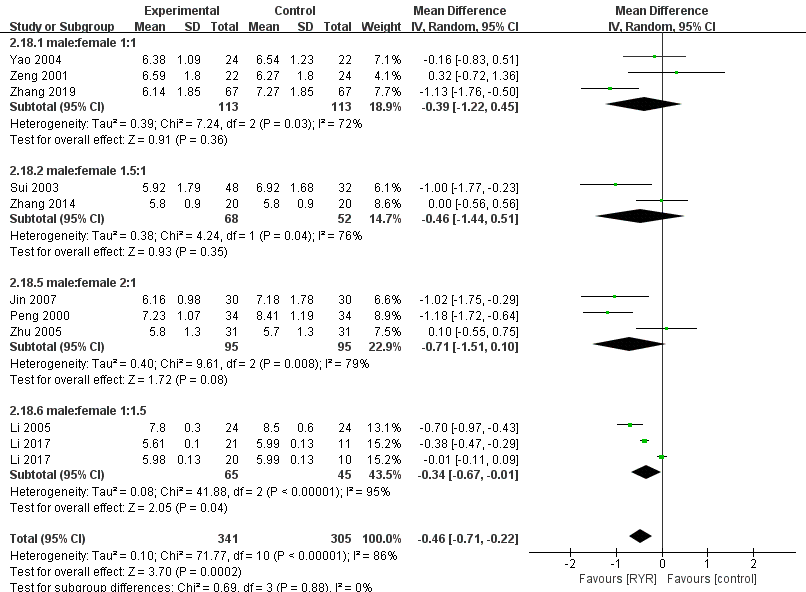

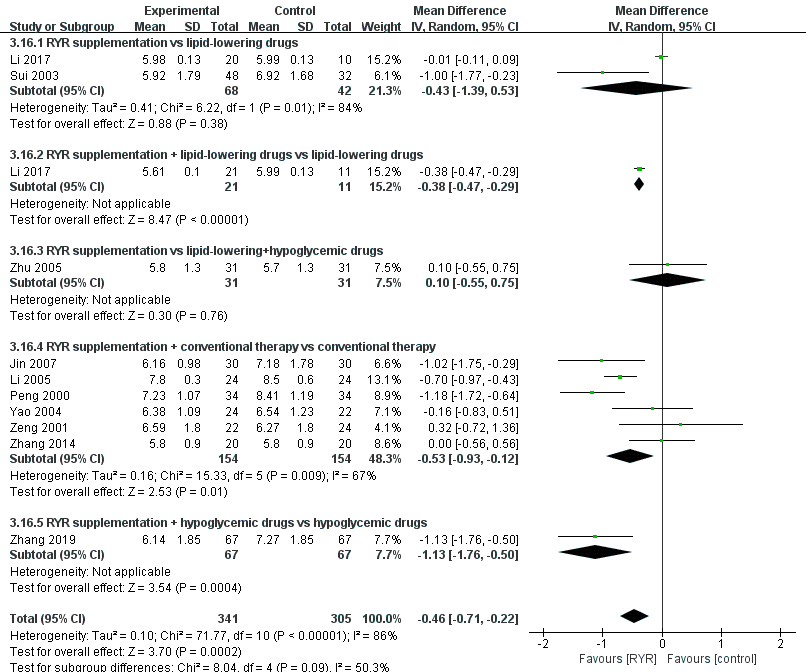


C D

Supplementary Figure S1 Forest plots of the subgroup analysis of FPG classified by (A) age, (B) intervention duration, (C) sex, and (D) intervention type


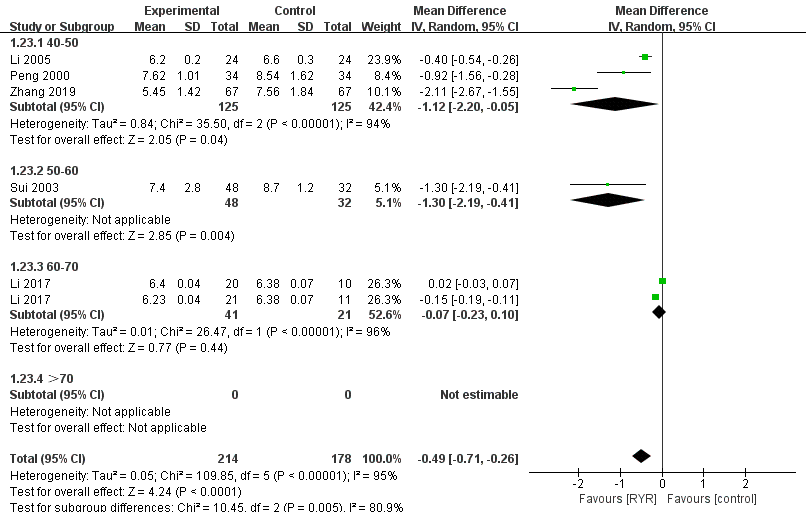

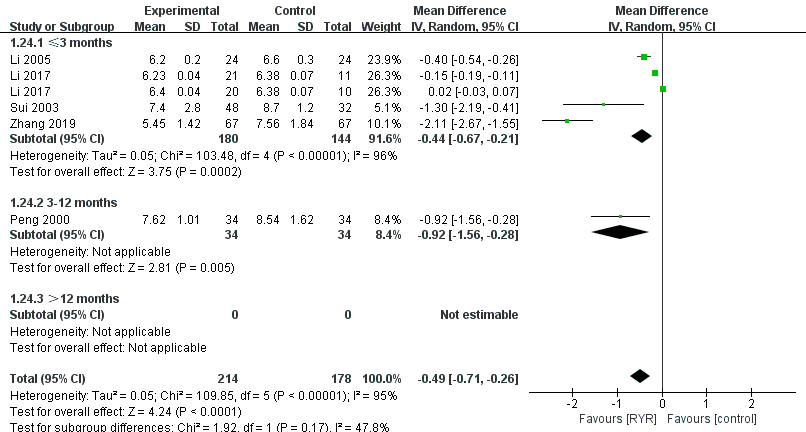


A B


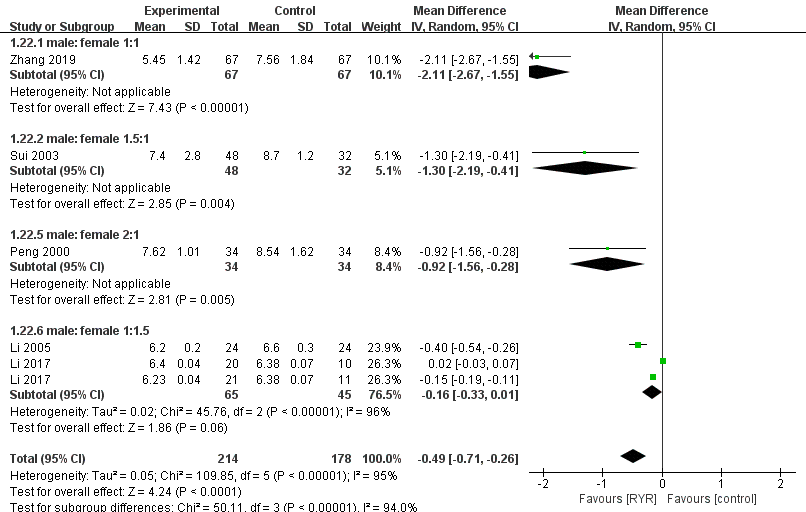

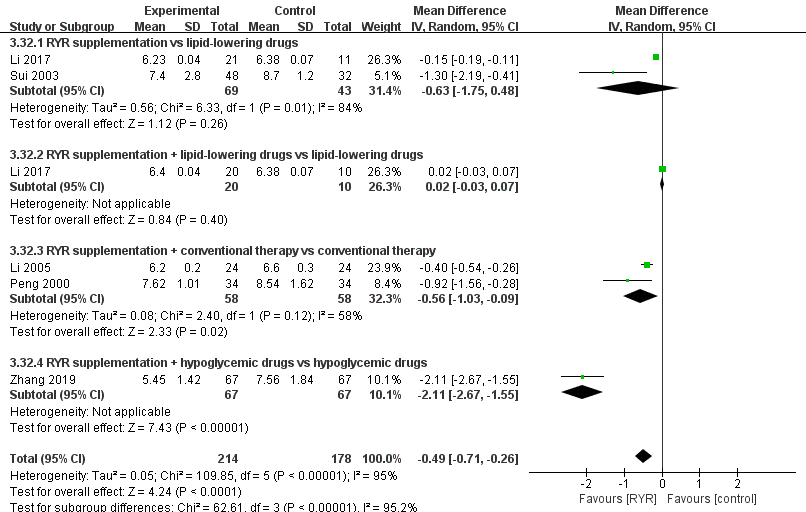


C D

Supplementary Figure S2 Forest plots of the subgroup analysis of HbA1c classified by (A) age, (B) intervention duration, (C) sex, and (D) intervention type


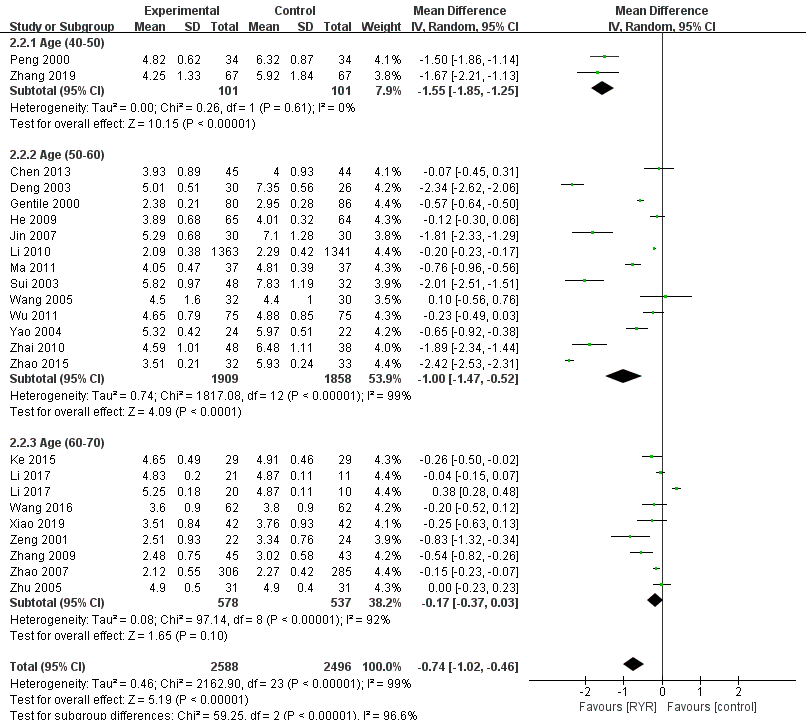

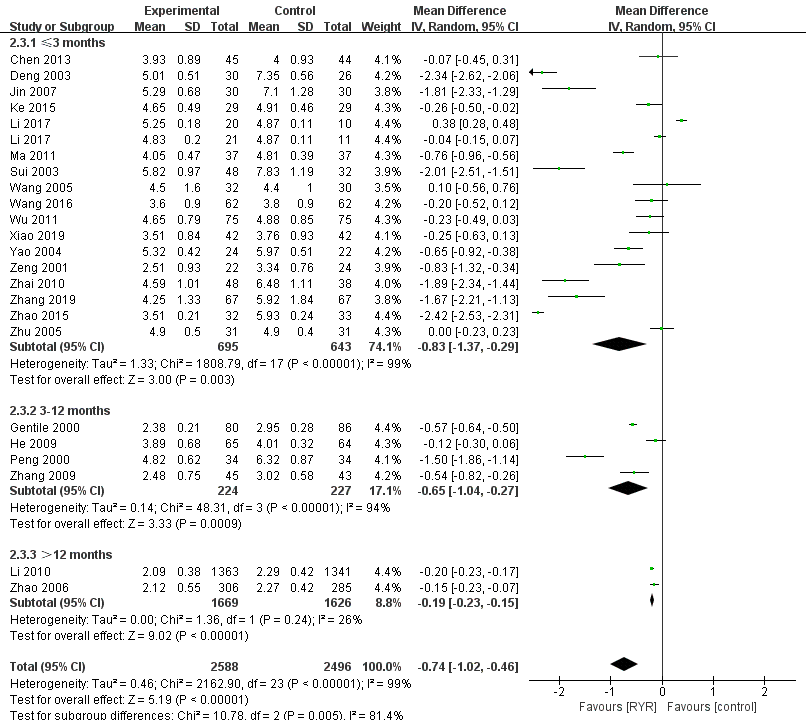


A B


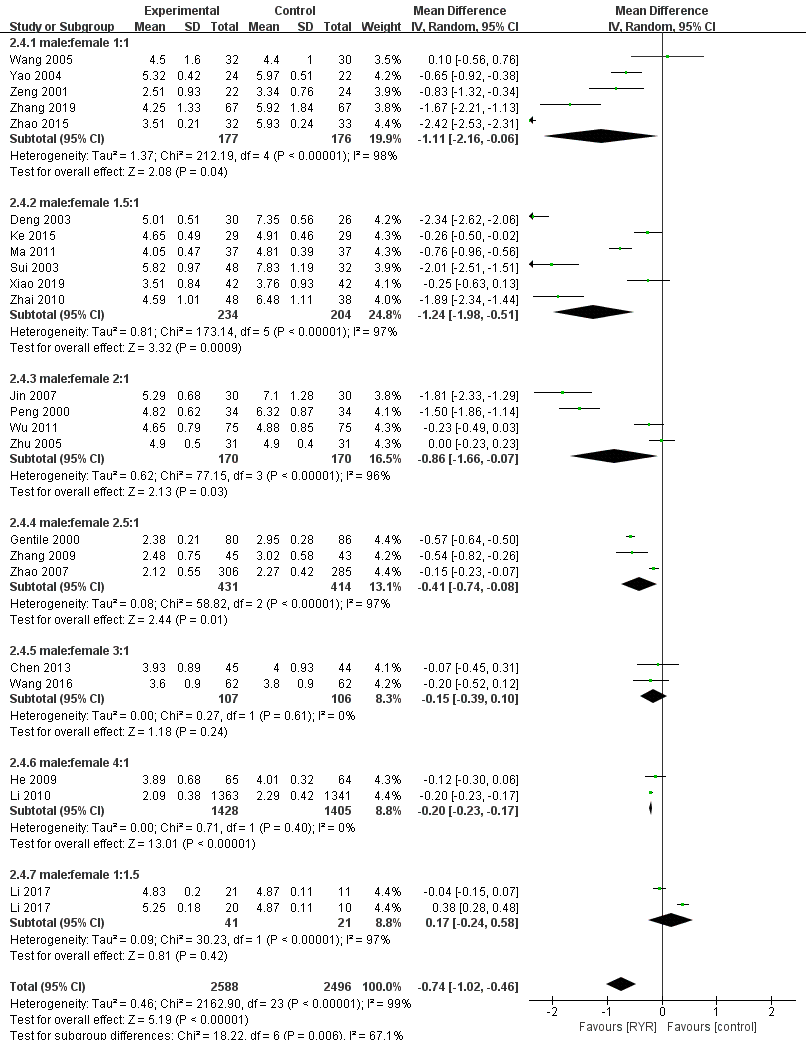

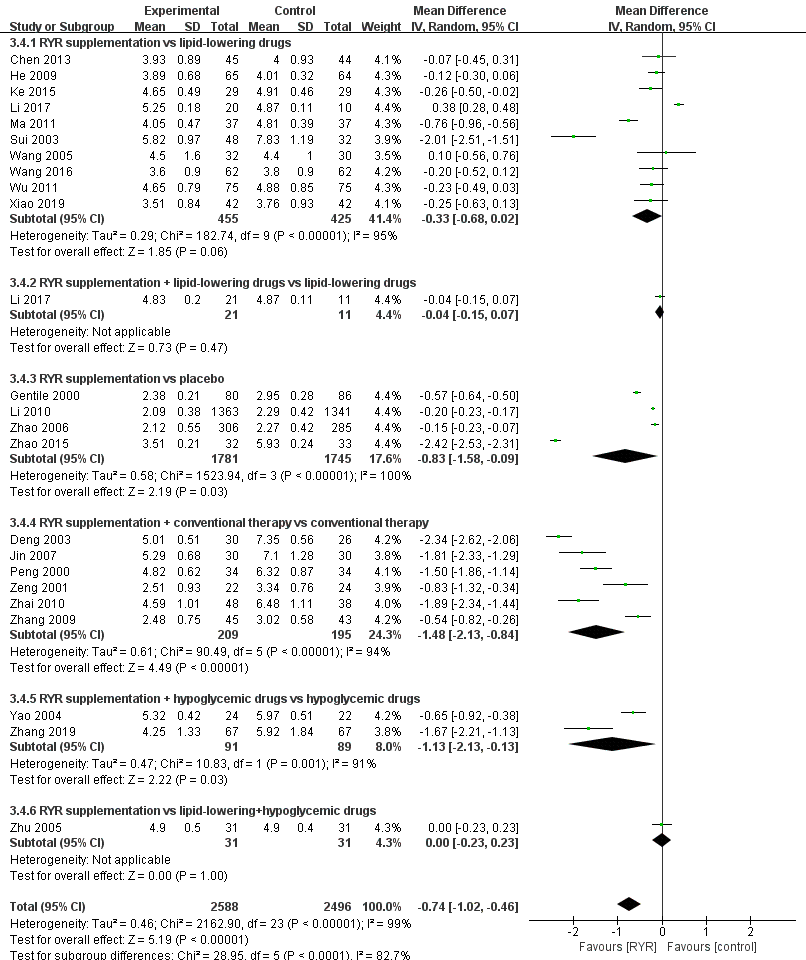


C D

Supplementary Figure S3 Forest plots of the subgroup analysis of TC classified by (A) age, (B) intervention duration, (C) sex, and (D) intervention type


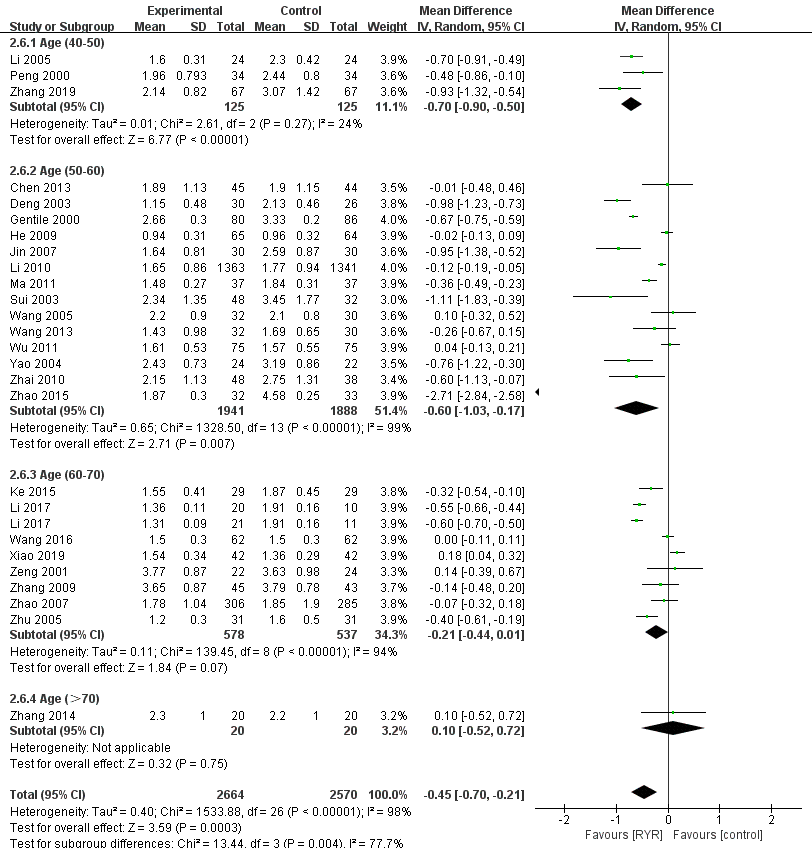

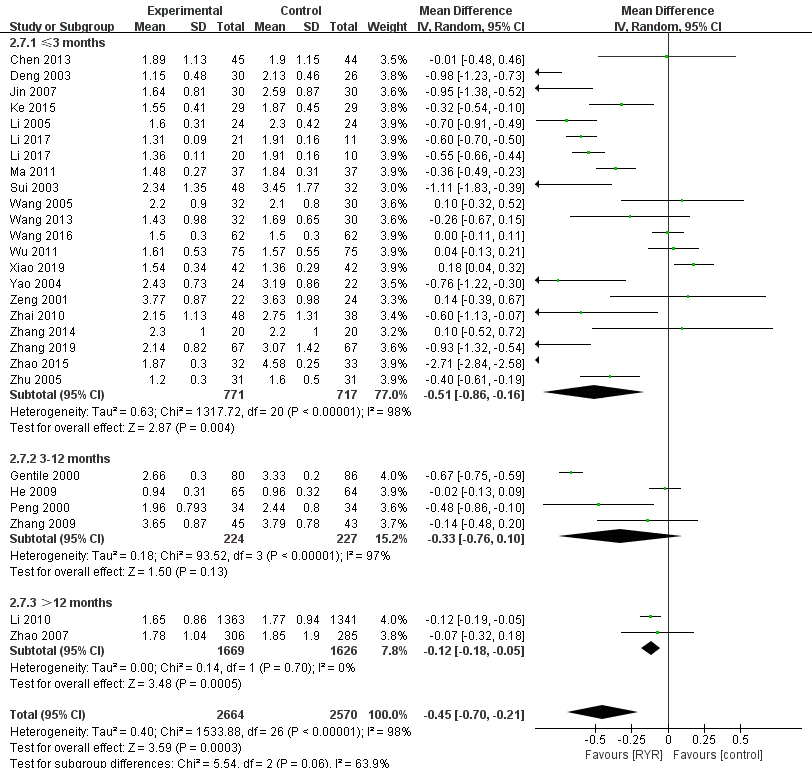


A B


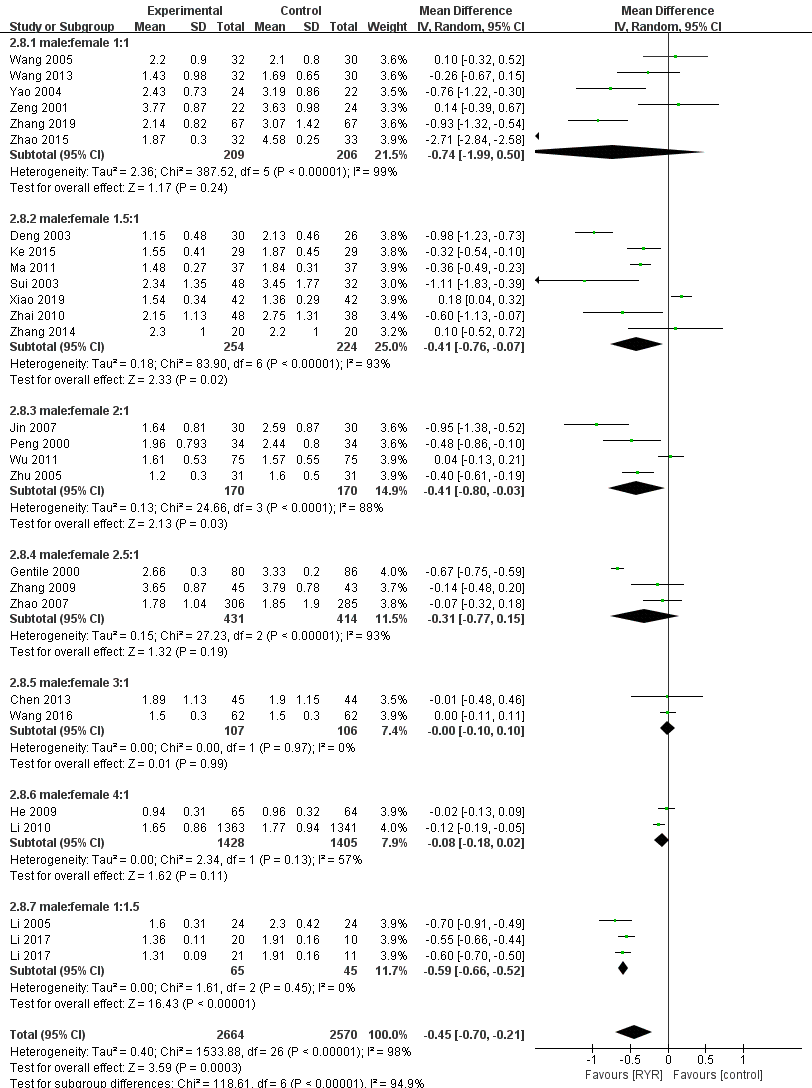

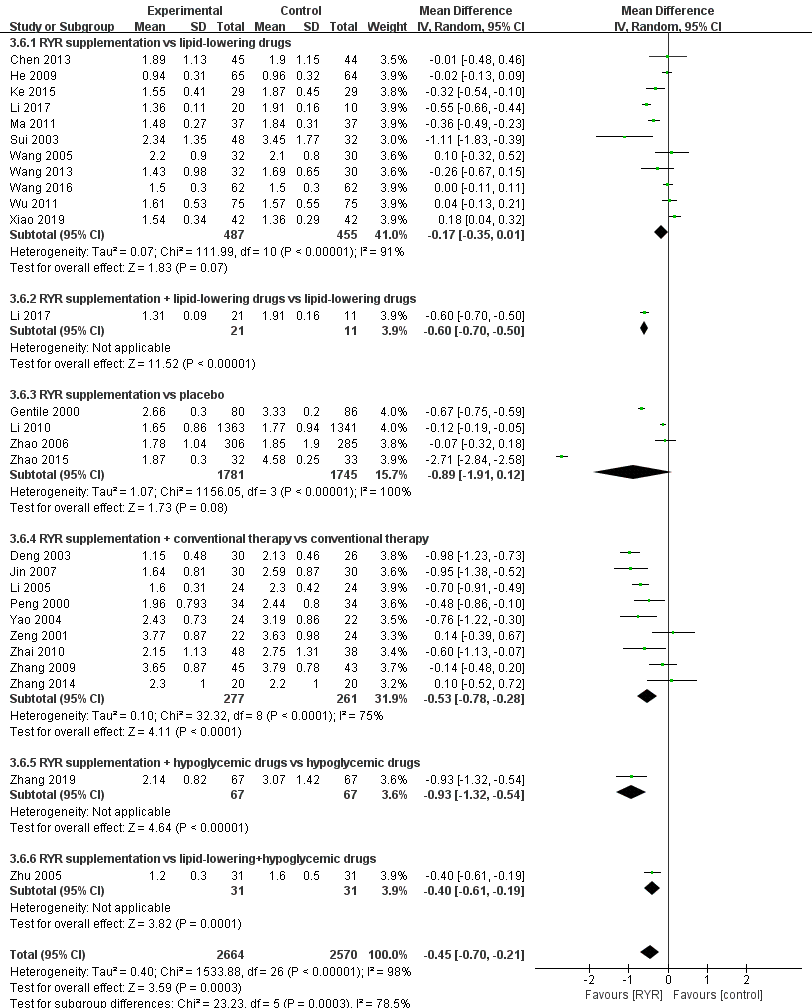


C D

Supplementary Figure S4 Forest plots of the subgroup analysis of TG classified by (A) age, (B) intervention duration, (C) sex, and (D) intervention type


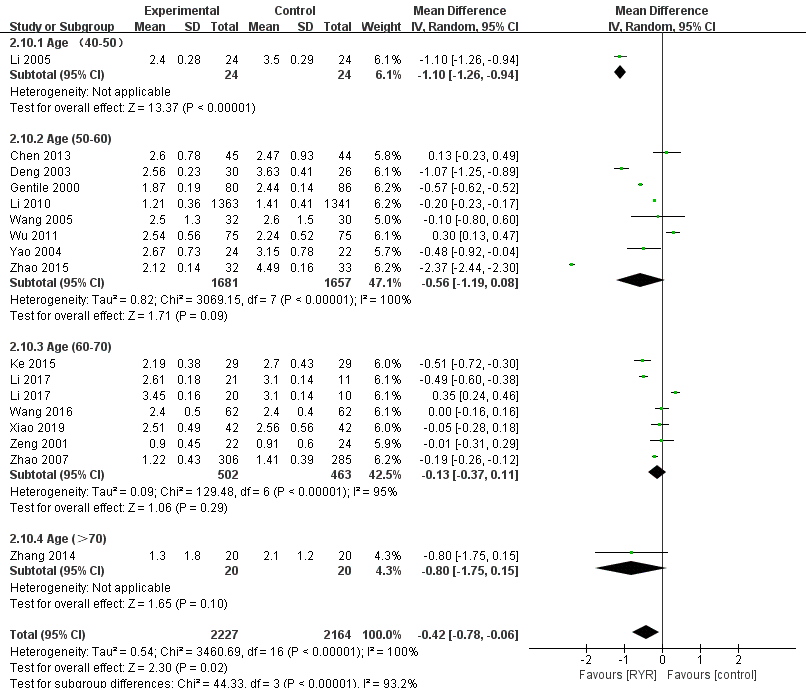

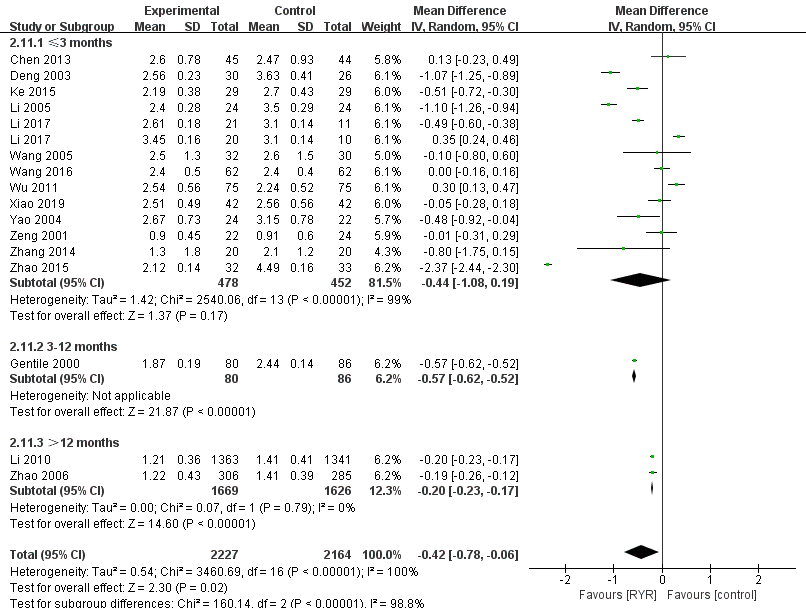


A B


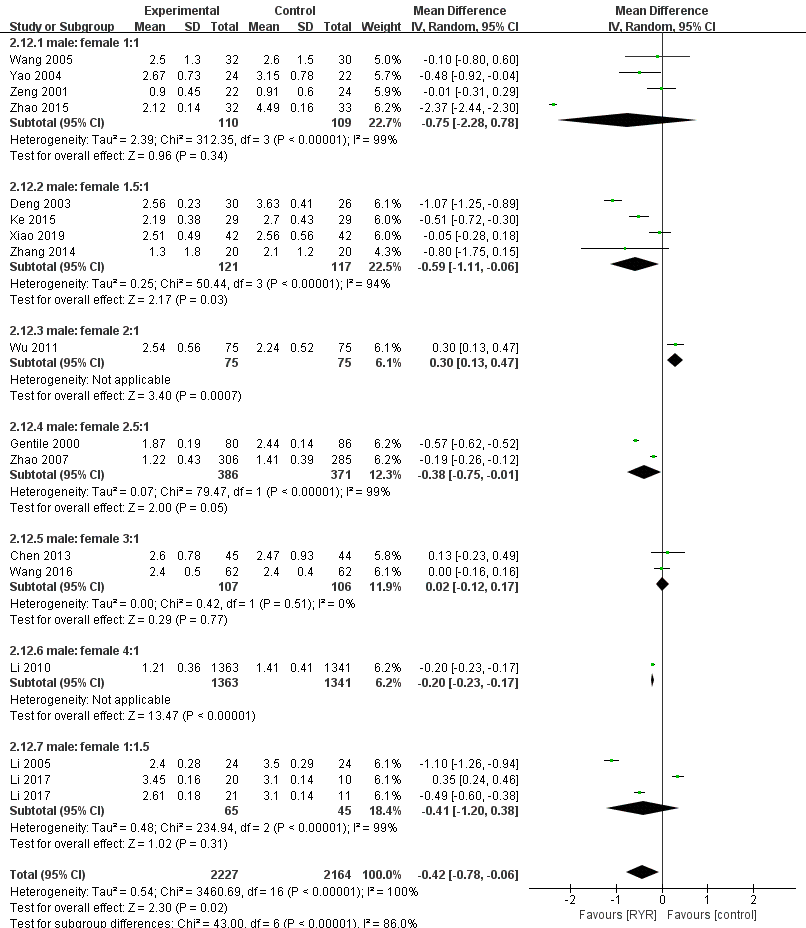

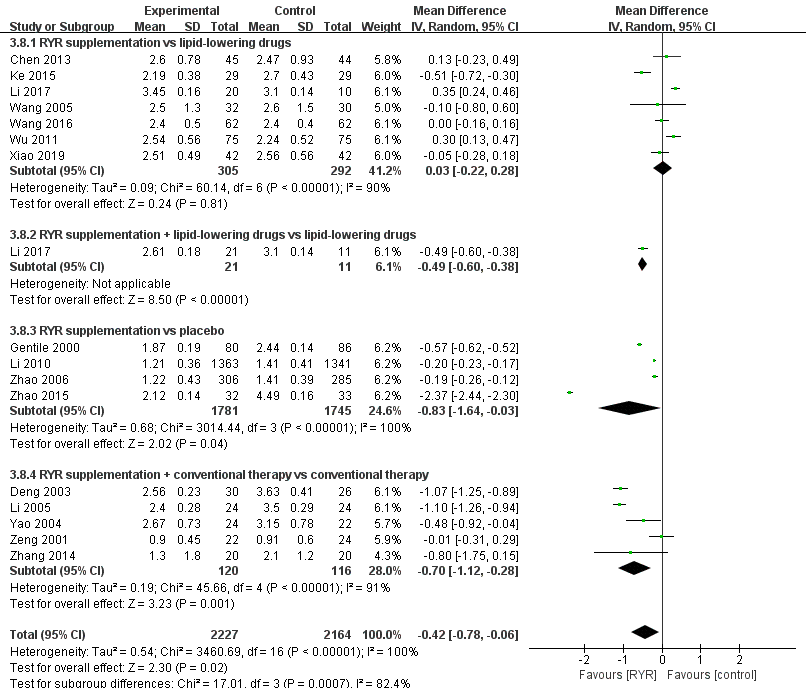


C D

Supplementary Figure S5 Forest plots of the subgroup analysis of LDL classified by (A) age, (B) intervention duration, (C) sex, and (D) intervention type


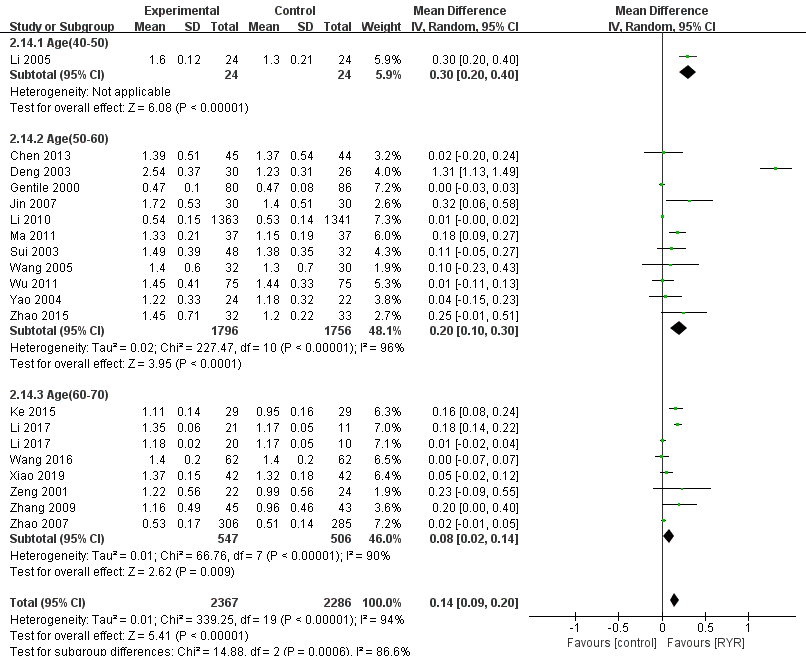

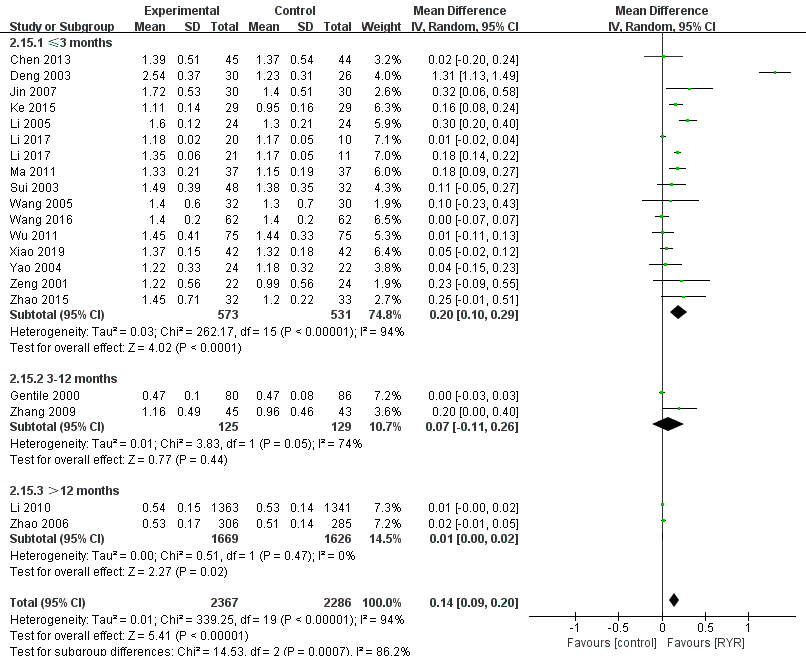


A B


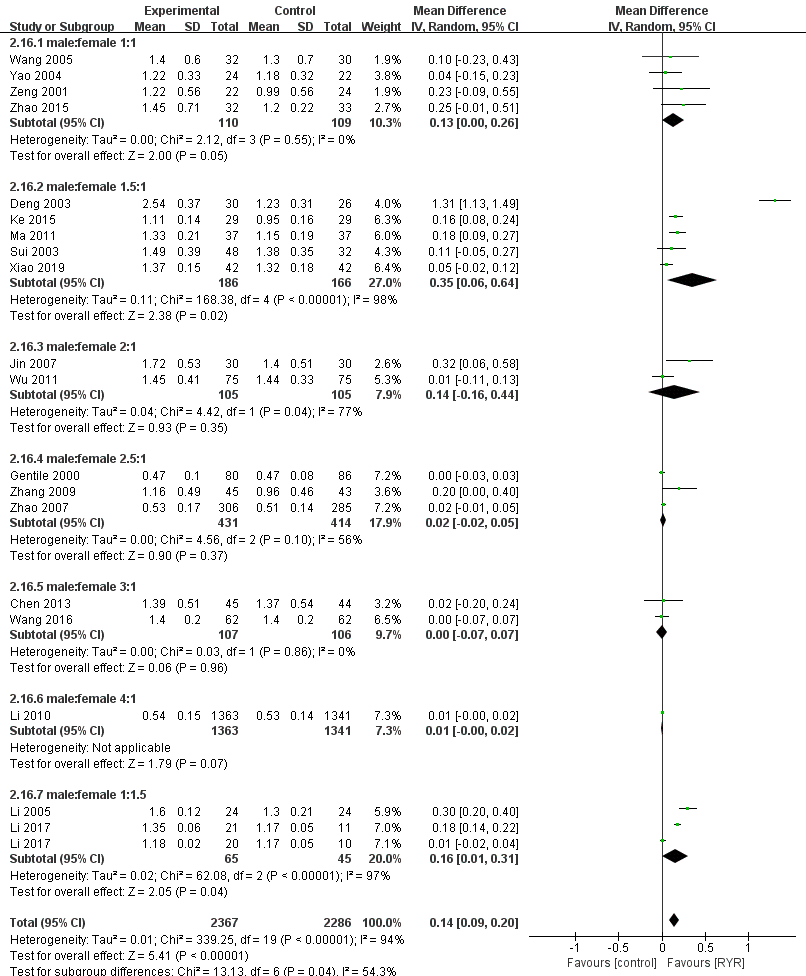

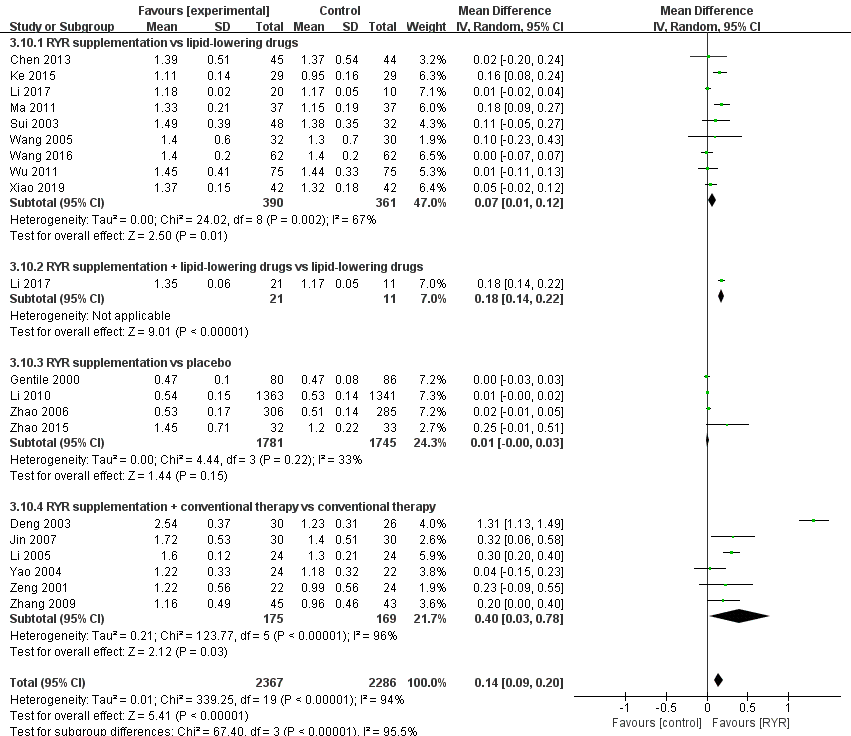


C D

Supplementary Figure S6 Forest plots of the subgroup analysis of HDL classified by (A) age, (B) intervention duration, (C) sex, and (D) intervention type


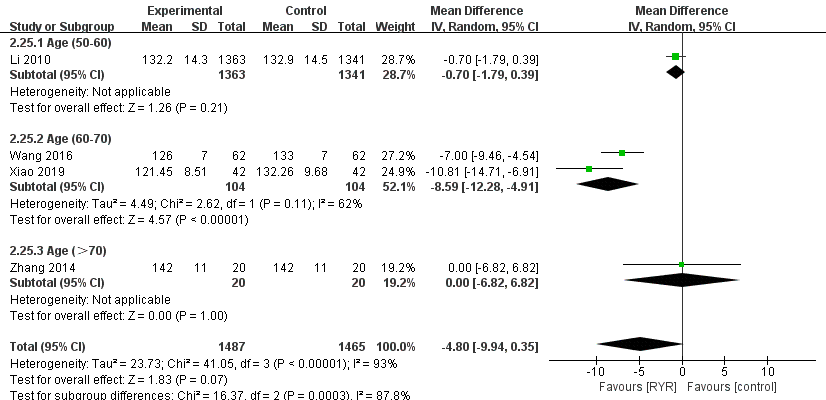

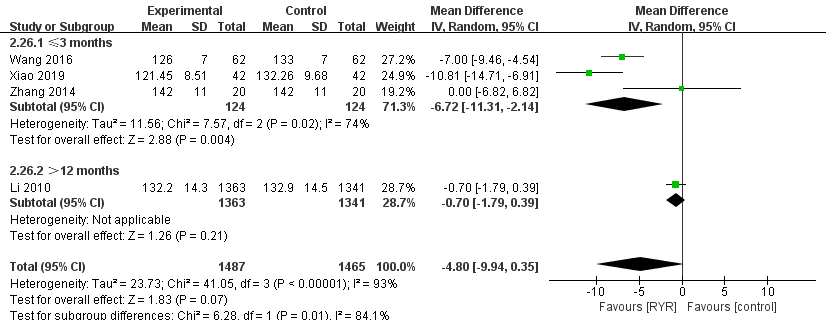


A B


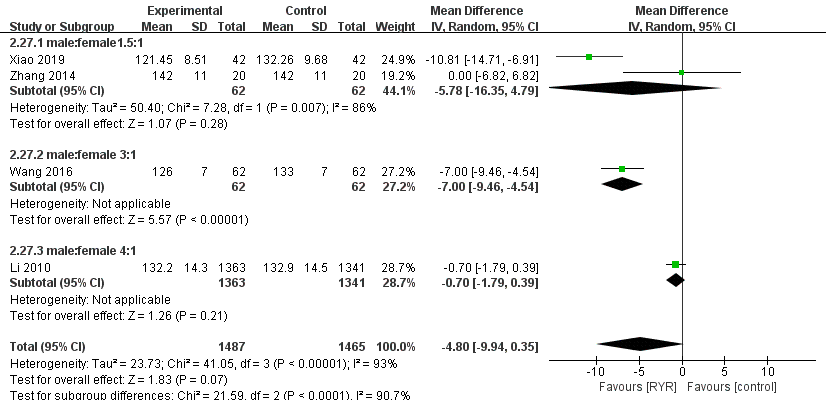

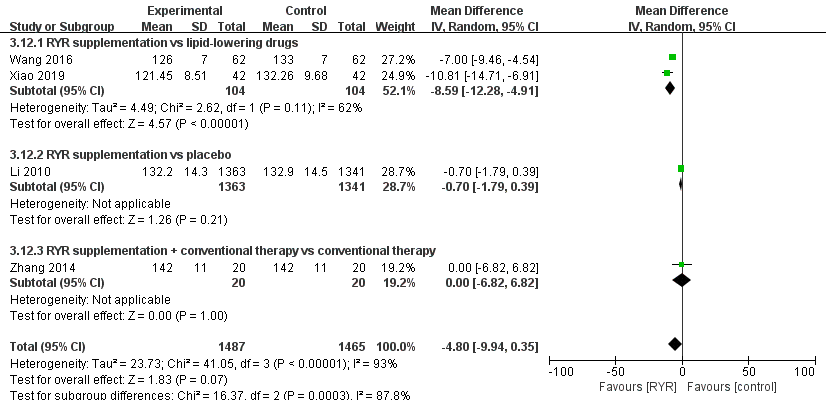


C D

Supplementary Figure S7 Forest plots of the subgroup analysis of SBP classified by (A) age, (B) intervention duration, (C) sex, and (D) intervention type


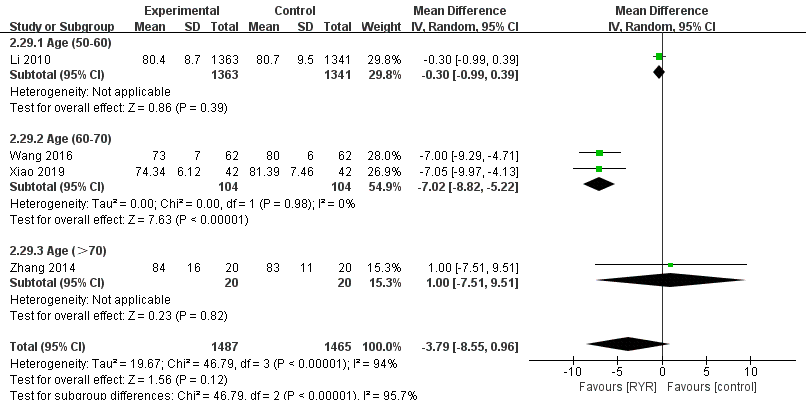

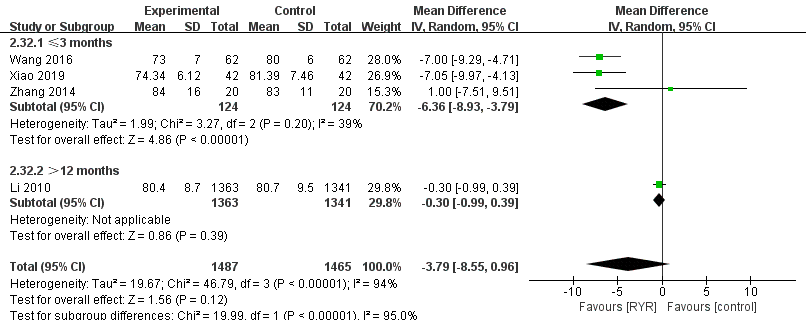


A B


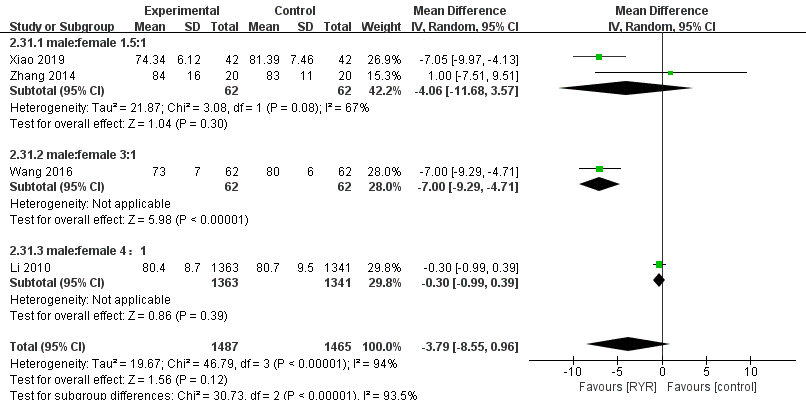

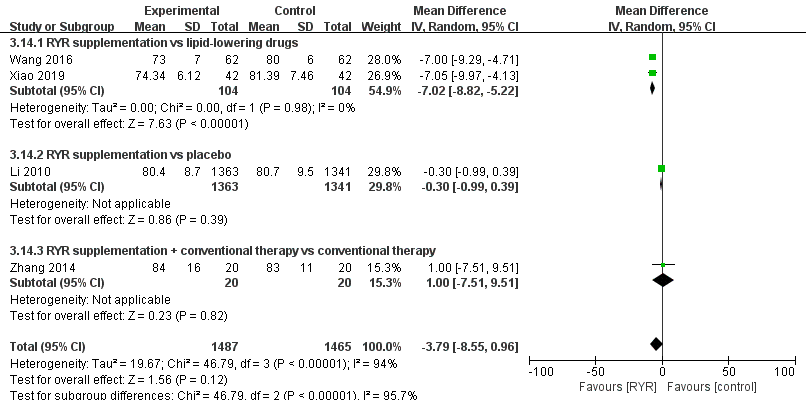


C D

Supplementary Figure S8 Forest plots of the subgroup analysis of DBP classified by (A) age, (B) intervention duration, (C) sex, and (D) intervention type
