# Supplementary material for: Red Yeast Rice Preparations Reduce Mortality, Major Cardiovascular Adverse Events, and Risk Factors for Metabolic Syndrome: A Systematic Review and Meta−analysis
Source: Front Pharmacol. 2022 Feb 21;13:744928. doi: 10.3389/fphar.2022.744928 (PMC8899821; doi:10.3389/fphar.2022.744928)
Supplement: Supplementary file 3 [file DataSheet1.docx]

Supplementary Material 1. Search strategy (Medline)

(((((((((((((("Metabolic Syndrome"[Mesh]) OR (((((((((((((((((((Metabolic Syndromes[Title/Abstract]) OR (Syndrome, Metabolic[Title/Abstract])) OR (Syndromes, Metabolic[Title/Abstract])) OR (Metabolic Syndrome X[Title/Abstract])) OR (Insulin Resistance Syndrome X[Title/Abstract])) OR (Syndrome X, Metabolic[Title/Abstract])) OR (Syndrome X, Insulin Resistance[Title/Abstract])) OR (Metabolic X Syndrome[Title/Abstract])) OR (Syndrome, Metabolic X[Title/Abstract])) OR (X Syndrome, Metabolic[Title/Abstract])) OR (Dysmetabolic Syndrome X[Title/Abstract])) OR (Syndrome X, Dysmetabolic[Title/Abstract])) OR (Reaven Syndrome X[Title/Abstract])) OR (Syndrome X, Reaven[Title/Abstract])) OR (Metabolic Cardiovascular Syndrome[Title/Abstract])) OR (Cardiovascular Syndrome, Metabolic[Title/Abstract])) OR (Cardiovascular Syndromes, Metabolic[Title/Abstract])) OR (Syndrome, Metabolic Cardiovascular[Title/Abstract])) OR (insulin resistance syndrome[Title/Abstract]))) OR ((((blood pressure[Title/Abstract]) OR (hypertension[Title/Abstract])) OR (hypertensive[Title/Abstract])) AND (((((((((((cholesterol[Title/Abstract]) OR (triglyceride[Title/Abstract])) OR (dyslipidemia[Title/Abstract])) OR (dyslipoproteinemia[Title/Abstract])) OR (lipid disorder[Title/Abstract])) OR (lipid metabolic disorders[Title/Abstract])) OR (hypercholesteremia[Title/Abstract])) OR (hyperlipidemias[Title/Abstract])) OR (hyperlipemia[Title/Abstract])) OR (lipidemia[Title/Abstract])) OR (Lipemia[Title/Abstract])))) OR ((((blood pressure[Title/Abstract]) OR (hypertension[Title/Abstract])) OR (hypertensive[Title/Abstract])) AND ((obesity[Title/Abstract]) OR (waist circumference[Title/Abstract])))) OR ((((blood pressure[Title/Abstract]) OR (hypertension[Title/Abstract])) OR (hypertensive[Title/Abstract])) AND ((glucose[Title/Abstract]) OR (hyperglycemia[Title/Abstract])))) OR ((((blood pressure[Title/Abstract]) OR (hypertension[Title/Abstract])) OR (hypertensive[Title/Abstract])) AND (proteinuria[Title/Abstract]))) OR ((((((((((((cholesterol[Title/Abstract]) OR (triglyceride[Title/Abstract])) OR (dyslipidemia[Title/Abstract])) OR (dyslipoproteinemia[Title/Abstract])) OR (lipid disorder[Title/Abstract])) OR (lipid metabolic disorders[Title/Abstract])) OR (hypercholesteremia[Title/Abstract])) OR (hyperlipidemias[Title/Abstract])) OR (hyperlipemia[Title/Abstract])) OR (lipidemia[Title/Abstract])) OR (Lipemia[Title/Abstract])) AND ((obesity[Title/Abstract]) OR (waist circumference[Title/Abstract])))) OR ((((((((((((cholesterol[Title/Abstract]) OR (triglyceride[Title/Abstract])) OR (dyslipidemia[Title/Abstract])) OR (dyslipoproteinemia[Title/Abstract])) OR (lipid disorder[Title/Abstract])) OR (lipid metabolic disorders[Title/Abstract])) OR (hypercholesteremia[Title/Abstract])) OR (hyperlipidemias[Title/Abstract])) OR (hyperlipemia[Title/Abstract])) OR (lipidemia[Title/Abstract])) OR (Lipemia[Title/Abstract])) AND ((glucose[Title/Abstract]) OR (hyperglycemia[Title/Abstract])))) OR ((((((((((((cholesterol[Title/Abstract]) OR (triglyceride[Title/Abstract])) OR (dyslipidemia[Title/Abstract])) OR (dyslipoproteinemia[Title/Abstract])) OR (lipid disorder[Title/Abstract])) OR (lipid metabolic disorders[Title/Abstract])) OR (hypercholesteremia[Title/Abstract])) OR (hyperlipidemias[Title/Abstract])) OR (hyperlipemia[Title/Abstract])) OR (lipidemia[Title/Abstract])) OR (Lipemia[Title/Abstract])) AND (proteinuria[Title/Abstract]))) OR (((obesity[Title/Abstract]) OR (waist circumference[Title/Abstract])) AND ((glucose[Title/Abstract]) OR (hyperglycemia[Title/Abstract])))) OR (((obesity[Title/Abstract]) OR (waist circumference[Title/Abstract])) AND (proteinuria[Title/Abstract]))) OR (((glucose[Title/Abstract]) OR (hyperglycemia[Title/Abstract])) AND (proteinuria[Title/Abstract]))) AND (("red yeast rice" [Supplementary Concept]) OR ((((((((((((((((((((((((((((((Red-yeast-rice[Title/Abstract]) OR (RYR[Title/Abstract])) OR (R-Y-R[Title/Abstract])) OR (Red yeast rice extract[Title/Abstract])) OR (Red-yeast-rice extract[Title/Abstract])) OR (Red rice yeast[Title/Abstract])) OR (Red-rice-yeast[Title/Abstract])) OR (Red rice[Title/Abstract])) OR (mold rice[Title/Abstract])) OR (Mould rice[Title/Abstract])) OR (Fermented rice[Title/Abstract])) OR (Went rice[Title/Abstract])) OR (Monacolin k[Title/Abstract])) OR (MonK[Title/Abstract])) OR (Cholestin[Title/Abstract])) OR (Monascus[Title/Abstract])) OR (Monascus Purpureus[Title/Abstract])) OR (Monascus Purpureus Went[Title/Abstract])) OR (Monacolin[Title/Abstract])) OR (Hong qu[Title/Abstract])) OR (Chinese red-yeast-rice dietary supplement[Title/Abstract])) OR (Xuezhikang[Title/Abstract])) OR (Zhi Tai[Title/Abstract])) OR (Red koji[Title/Abstract])) OR (Xuezhikang tablet[Title/Abstract])) OR (Xuezhikang Capsule[Title/Abstract])) OR (XZK[Title/Abstract])) OR (Zhibituo[Title/Abstract])) OR (Hypocol blood lipid wenstardin[Title/Abstract])) OR (Mevinolin[Title/Abstract])))) AND (("Randomized Controlled Trial" [Publication Type]) OR ((((((((((clinical trial[Title/Abstract]) OR (controlled trial[Title/Abstract])) OR (randomized controlled trial[Title/Abstract])) OR (randomised controlled trial[Title/Abstract])) OR (randomized[Title/Abstract])) OR (randomised[Title/Abstract])) OR (trial[Title/Abstract])) OR (controlled clinical trial[Title/Abstract])) OR (clinical study[Title/Abstract])) OR (RCT[Title/Abstract])))) NOT ((((animal[Title/Abstract]) OR (mice[Title/Abstract])) OR (mouse[Title/Abstract])) OR (rabbit[Title/Abstract]))
